# Enterohemorrhagic *Escherichia coli* Tir inhibits TAK1 activation and mediates immune evasion

**DOI:** 10.1080/22221751.2019.1620589

**Published:** 2019-05-25

**Authors:** Ruixue Zhou, Zijuan Chen, Doudou Hao, Yu Wang, Yihua Zhang, Xianfu Yi, Liang-Dong Lyu, Haipeng Liu, Quanming Zou, Yiwei Chu, Baoxue Ge, Dapeng Yan

**Affiliations:** a Department of Immunology, School of Basic Medical Sciences & Shanghai Public Health Clinical Center, Key Laboratory of Medical Molecular Virology of MOE/MOH, Fudan University, Shanghai, People’s Republic of China; b Department of Microbiology and Biochemical Pharmacy, National Engineering Research Centre of Immunological Products, College of Pharmacy, Army Medical University, Chongqing, People’s Republic of China; c School of Biomedical Engineering, Tianjin Medical University, Tianjin, People’s Republic of China; d Shanghai Key Laboratory of Tuberculosis, Shanghai Pulmonary Hospital, Tongji University School of Medicine, Shanghai, People’s Republic of China; e Department of Microbiology and Immunology, Tongji University School of Medicine, Shanghai, People’s Republic of China

**Keywords:** EHEC, Tir, ITIM, SHP-1, TAK1, immune evasion

## Abstract

Many pathogens infect hosts through various immune evasion strategies. However, the molecular mechanisms by which pathogen proteins modulate and evade the host immune response remain unclear. Enterohemorrhagic *Escherichia coli* (EHEC) is a pathological strain that can induce mitogen-activated protein (MAP) kinase (Erk, Jnk and p38 MAPK) and NF-κB pathway activation and proinflammatory cytokine production, which then causes diarrheal diseases such as hemorrhagic colitis and hemolytic uremic syndrome. Transforming growth factor β-activated kinase-1 (TAK1) is a key regulator involved in distinct innate immune signalling pathways. Here we report that EHEC translocated intimin receptor (Tir) protein inhibits the expression of EHEC-induced proinflammatory cytokines by interacting with the host tyrosine phosphatase SHP-1, which is dependent on the phosphorylation of immunoreceptor tyrosine-based inhibition motifs (ITIMs). Mechanistically, the association of EHEC Tir with SHP-1 facilitated the recruitment of SHP-1 to TAK1 and inhibited TAK1 phosphorylation, which then negatively regulated K63-linked polyubiquitination of TAK1 and downstream signal transduction. Taken together, these results suggest that EHEC Tir negatively regulates proinflammatory responses by inhibiting the activation of TAK1, which is essential for immune evasion and could be a potential target for the treatment of bacterial infection.

## Introduction

Humans have a sophisticated immune system to fight against pathogens. However, many pathogens employ unique strategies to evade, inhibit, or manipulate the innate immune response, leading to chronic infection [1]. These strategies include infecting immune cells or delitescence, changing self-structures to subvert pathogen recognition, and inhibiting host signal transduction by structural or non-structural components.

Immune responses are regulated by immune receptors that contain either an immunoreceptor tyrosine-based activation motif (ITAM) or immunoreceptor tyrosine-based inhibitory motif (ITIM) [2–4]. Tyrosine phosphorylation in the ITIMs creates binding sites for SH2-containing phosphatases, such as SHP-1 [5–7]. SHP-1 is an intracellular protein tyrosine phosphatase, which may negatively regulate toll-like receptor mediated proinflammatory cytokine production by inhibiting the activation of transcription factor NF-κB and mitogen-activated protein kinase [8,9].

TAK1 was originally identified as a member of the transforming growth factor-β (TGF-β)-activated MAPK kinase kinase (MKKK) family [10]. Multiple lines of evidence have shown that TAK1 is involved in distinct signalling pathways induced by various stimuli, such as environmental stress [11], inflammatory cytokines [12], lipopolysaccharide (LPS) [13], and TGF-β [14]. Phosphorylation of TAK1 at Thr-187, Ser-192 and Ser-412 is required for its activation [15,16]. Once activated, TAK1 can activate several downstream cellular signalling cascades, such as p38 MAPK, JNK, IKK, and Akt [17], leading to the translocation of the transcription factors NF-κB and AP-1 [18,19] from the cytoplasm to nucleus and subsequent production of proinflammatory cytokines.

EHEC, which is associated with the consumption of contaminated food and water, constitutes a significant human health risk and may cause acute gastroenteritis, bloody diarrhea and hemorrhagic colitis. More than 10% of patients develop severe complications with 5% mortality [20]. EHEC generates attaching and effacing (AE) lesions to colonize the intestine, damage the epithelium, and promote diarrheal illnesses [21,22]. The translocated intimin receptor (Tir) from EHEC is an important virulence protein that is injected into the plasma membrane of host cells via a type III secretion system (T3SS) [23–26]. Proteases produced by EHEC and the prevalent human commensal *Bacteroides thetaiotaomicron* can modulate T3SS function by cleaving proteins in the EHEC T3SS translocon [27]. Our previous results showed that the enteropathogenic *E. coli* (EPEC) Tir recruits SHP-1 to inhibit tumour necrosis factor (TNF) receptor-associated factor 6 (TRAF6) ubiquitination [28–30]. Increasing evidence shows that these T3SS-bearing pathogens share similarities to target key cellular functions such as the cell cytoskeleton, trafficking, cell death/survival, and signalling pathways such as NF-κB and MAPK [31]. However, whether EHEC Tir regulates the immune response to EHEC infection and the mechanisms involved in this process remains unknown. Therefore, we aimed to determine whether EHEC Tir might have important roles in regulating and evading the host immune response.

## Materials and methods

### Mice, bacterial growth, and cell culture

*Ptpn6*^me-v/me-v^ (000811; Jackson Laboratories) mice on C57BL/6 background were maintained in clean and comfortable conditions at the Laboratory Animal Center of Fudan University. 3-4-week-old male mice were used in the experiments and littermates were used as controls. All animal studies were approved by the Institutional Animal Care and Use Committee of Fudan University.

EDL933 and EDL933 ΔTir were grown at 37°C in Luria–Bertani broth (10 g/L tryptone, 5 g/L yeast extract and 10 g/L NaCl). The bacterial mutants of Tir encoded in pK184 strains were grown in Luria–Bertani broth supplemented with 50 ng/ul kanamycin. Before infection, EDL933 and its mutants were propagated in Dulbecco’s modified Eagle’s medium (DMEM; HyClone) supplemented with 10 mM HEPES (pH 7.4) for 15 h at 37°C in 5% CO_2_.

HEK293 T cells, RAW264.7 cells and mouse primary peritoneal macrophages were cultured in DMEM supplemented with 10% (v/v) FBS (Gibco) and 100 U/ml penicillin and streptomycin. The HeLa cells were maintained in RPMI 1640 medium supplemented with 10% (v/v) FBS (Gibco) and 100 U/ml penicillin and streptomycin.

### Reagents and antibodies

**Table:** 

REAGENT or RESOURCE	SOURCE	DENTIFIER
monoclonal mouse anti-Flag M2 Affinity Gel	Sigma	A2220
rabbit anti-Flag	Sigma	F7425
rabbit anti-HA	Sigma	H6908
monoclonal mouse anti-HA	Sigma	H9658
TAK1 (M-579) rabbit polyclonal antibody	Santa Cruz Biotechnology	sc-7162
mouse TAK1 (C-9) monoclonal antibody	Santa Cruz Biotechnology	sc-7967
rabbit *p*-TAK1 (T187) antibody	Cell Signaling Technology	4536S
rabbit *p*-TAK1(S412) antibody	Cell Signaling Technology	9339S
rabbit *p*-TAK1(Ser192) polyclonal antibody	Santa Cruz Biotechnology	sc-130219
rabbit ubiquitin antibody	Cell Signaling Technology	3933S
rabbit K63-linkage specific polyubiquitin (D7A11) monoclonal antibody	Cell Signaling Technology	5621S
rabbit K48-linkage specific polyubiquitin (D9D5) monoclonal antibody	Cell Signaling Technology	8081S
rabbit IκB-alpha(44D4) monoclonal antibody	Cell Signaling Technology	4812S
rabbit *p*-p44/42 MAPK (*p*-Erk) (T202/Y204) antibody	Cell Signaling Technology	9101S
rabbit *p*-p38 MAPK (T180/Y182) monoclonal antibody	Cell Signaling Technology	9215S
rabbit *p*-p65 (S536) monoclonal antibody	Cell Signaling Technology	3033S
rabbit *p*-Jnk (T183/Y185) antibody	Cell Signaling Technology	9251S
rabbit *p*-IKKα/β (Ser176/180) (16A6) antibody	Cell Signaling Technology	2697S
protein A/G PLUS-Agarose	Santa Cruz Biotechnology	sc-2003
rabbit anti-SHP-1	Abcam	ab55356
rabbit anti-phospho tyrosine	Abcam	ab179530
Alexa Fluor^R^ 594 goat anti-rabbit IgG(H + L)	Invitrogen	SA11012s
Alexa Fluor^R^ 488 goat anti-mouse IgG (H + L)	Invitrogen	SA11001s

### Plasmid construction

Flag-EDL933 Tir (wild-type (WT), Y490F, Y519F, Y490F, and Y519F) were constructed using standard molecular biology techniques, and the cDNAs were inserted into the Flag-pcDNA3.0 vector. Glutathione S-transferase (GST)-EDL933 Tir (WT, Y490F, Y519F, Y490F, and Y519F) were inserted into pGEX4T-1 from the Flag-tagged plasmids described above. SHP-1, SHP-1 (C453S), SHP-2, TAK1, and HA-EDL933 Tir were inserted into HA-pcDNA3.0. The primers are listed in Supplementary Table 1.

#### Transfection and immunoprecipitation

HEK293 T cells were transfected using the PEI method. Pervanadate (0.1 mM sodium orthovanadate and 10 mM H_2_O_2_) was added for 20 min at 37°C (later 48 h) before cells were lysed in lysis buffer (Beyotime) supplemented with 1% Protease Inhibitor Cocktail (B14002; Selleck.cn), 1 mM NaF and 1 mM Na_3_VO_4_. For ubiquitination detection in co-immunoprecipitation experiments, cell lysates were treated with 1% SDS and boiled at 95°C for 5 min prior to immunoprecipitation. Cell lysates were rotated overnight at 4 °C with anti-Flag M2 Affinity Gel. The sepharose samples were centrifuged, washed three times with washing buffer (PBS, 1% Triton X-100), and boiled with SDS loading buffer for 10 min and later analyzed by immunoblot.

#### Confocal microscopy

HeLa cells were transfected by Lipofectamine 2000 (11668–019, Invitrogen). The cells were infected for 4 h with EDL933 (ΔTir + HA-Tir) or EDL933ΔTir, treated by standard immunofluorescent staining techniques and examined with a Leica confocal microscope.

#### GST pull-down

The GST-tagged EDL933 Tir plasmids and its mutants Y490F, Y519F, and Y490F/Y519F were transformed into TKB1 and induced to express either phosphorylated WT EDL933 Tir or its point mutants by IPTG. Then, recombinant proteins were purified as GST fusion proteins and incubated with SHP-1 purified from BL-21 strain or mouse primary peritoneal macrophages. The mixture samples were centrifuged, washed three times with washing buffer (PBS, 1% Triton X-100), and boiled with SDS loading buffer for 10 min and later analyzed by immunoblot.

#### RNA interference

SHP-1 siRNA (5′-GAGAUGGCACCAUCAUCCACCUUAA-3’) was used for inhibiting the expression of endogenous SHP-1 and control siRNA (5’-UUCUCCGAACGUGUCACGUTT-3’) was used as a control siRNA. They were transfected into RAW264.7 cells by the Amaxa program D-032 with the Cell Line Nucleofector Kit.

#### Quantitative RT–PCR

Total RNA was extracted to measure the mRNA abundance of the indicated genes by standard molecular biology techniques. Briefly, Total RNA was extracted with 1 ml of TRIzol reagent according to the manufacturer's instructions (Invitrogen). Next, 1 μg total RNA was reverse-transcribed with the ReverTra Ace qPCR RT Kit (FSQ-101) according to the manufacturer's instructions (Toyobo). A LightCycler (LC480; Roche) and a SYBR RT–PCR kit (QPK-212; Toyobo) were used for quantitative real-time RT–PCR analysis. The data shown are the relative abundances of the indicated mRNAs derived from RAW264.7 or mouse primary peritoneal macrophages normalized to GAPDH. Gene-specific primer sequences are described in Supplementary Table 1.

#### RNA sequencing

A total amount of 1.5 μg RNA was used as input for sample preparations. Sequencing libraries were generated using the NEBNext® UltraTM RNA Library Prep Kit for Illumina® (NEB, USA). The clustering of the index-coded samples was performed on a cBot Cluster Generation System using the HiSeq 4000 PE Cluster Kit (Illumia) and the samples were sequenced on an Illumina Hiseq 4000 platform. In this process, paired-end reads of 150 bp were generated.

#### Oral infection, CFU counts, histology, and immunohistochemical analysis

3-4-week-old male C57Bl/6J mice were pretreated for 24 h with streptomycin and fasted for 12 h before the oral inoculation. Simultaneously, 1 × 10^9^ colony forming units (CFU) EDL933 and its ΔTir mutants were prepared and orally gavaged for each mouse. Fecal specimens, colons and spleens were collected, weighed, homogenized in PBS and plated on MacConkey agar plates for CFU analysis. Some colon and spleen tissues were retained for qPCR to detect cytokine production, and the other colons were fixed in 4% (v/v) paraformaldehyde for evaluation of the tissue pathology by Hematoxylin and eosin (H&E) staining and F4/80, CD3, CD19-specific immunohistochemistry.

## Results

### Tir inhibits immune responses to EHEC in vivo

**Figure 1.**
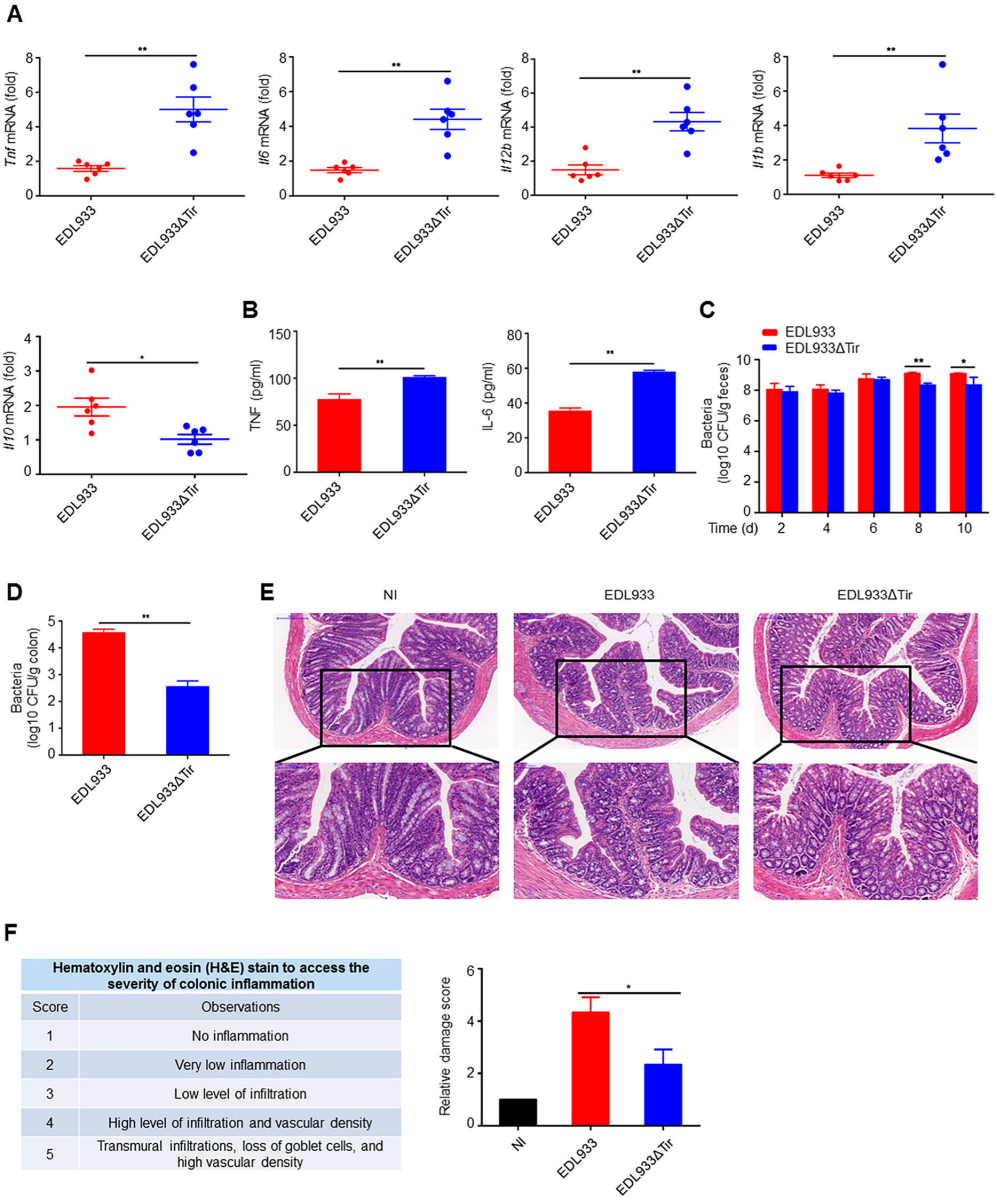
Tir inhibits immune responses to EHEC *in vivo.* a Quantitative RT–PCR analysis of *Tnf*, *Il6*, *Il12b,* or *Il1b* mRNA in colon from 3-4-week-old mice (*n* = 6 per group) infected orally with 1 × 10^9^ CFU of EDL933 or EDL933ΔTir and sacrified at fourteenth day. b ELISA analysis of TNF or IL-6 from mouse serum in **a**. **c-d** Bacterial load in the feces (**c**) or colon (**d**) of mice infected as in **a** at the indicated times. **e** Histopathology of colons of mice left uninfected (NI) or infected as in **a**. Outlined areas at top are enlarged below. Original magnification, ×10 (top) or ×20 (bottom). **f** Hematoxylin and eosin (H&E) stain to access the severity of colonic inflammation. Data are representative of at least two independent experiments (mean ± SEM in **a-d, f**). *p < 0.05 and **p < 0.01 (Student’s *t*-test).

The attachment of EHEC to its host cell is triggered by the intracellular interaction of its translocated effector proteins Tir and EspFU. The Asn-Pro-Tyr motif of Tir is critical for this interaction [32]. In order to determine whether the EHEC Tir protein inhibits the innate immune response *in vivo*, we constructed a mouse model where the mice were orally infected with EDL933 or EDL933ΔTir EHEC strains. We found that EDL933 induced a lower production of proinflammatory cytokines *Tnf, Il6, Il12b* and *Il1b* and a higher production of anti-inflammatory cytokines *Il10* than EDL933ΔTir in mouse colons (Figure 1a) and spleens (Supplemental Figure 1a) by quantitative real-time PCR (qPCR). In addition, EDL933-induced serum TNF and IL-6 were lower than the EDL933ΔTir strain did (Figure 1b). We next detected the amount of bacteria in mouse feces and colons in the two groups. The number of bacterial CFUs from the feces (Figure 1c) and colons (Figure 1d) infected with the EDL933 strain was significantly higher than those infected with the EDL933ΔTir strain. Moreover, mice infected with EDL933 had a slower weight gain than mice infected with the EDL933ΔTir strain (Supplemental Figure 1b). Histological examination revealed that mice infected with EDL933 had more extensive neutrophil inflammatory infiltrates and structural disruption of the colonic epithelium (Figure 1e-f). F4/80, CD3, and CD19-specific immunohistochemistry results show that mice infected with EDL933 had more macrophages, T cells and B cells, respectively (Supplemental Figure 1c). These results suggest that Tir may inhibit the intestinal immune response to EDL933 infection.

### EHEC Tir inhibits cytokine production in vitro

**Figure 2.**
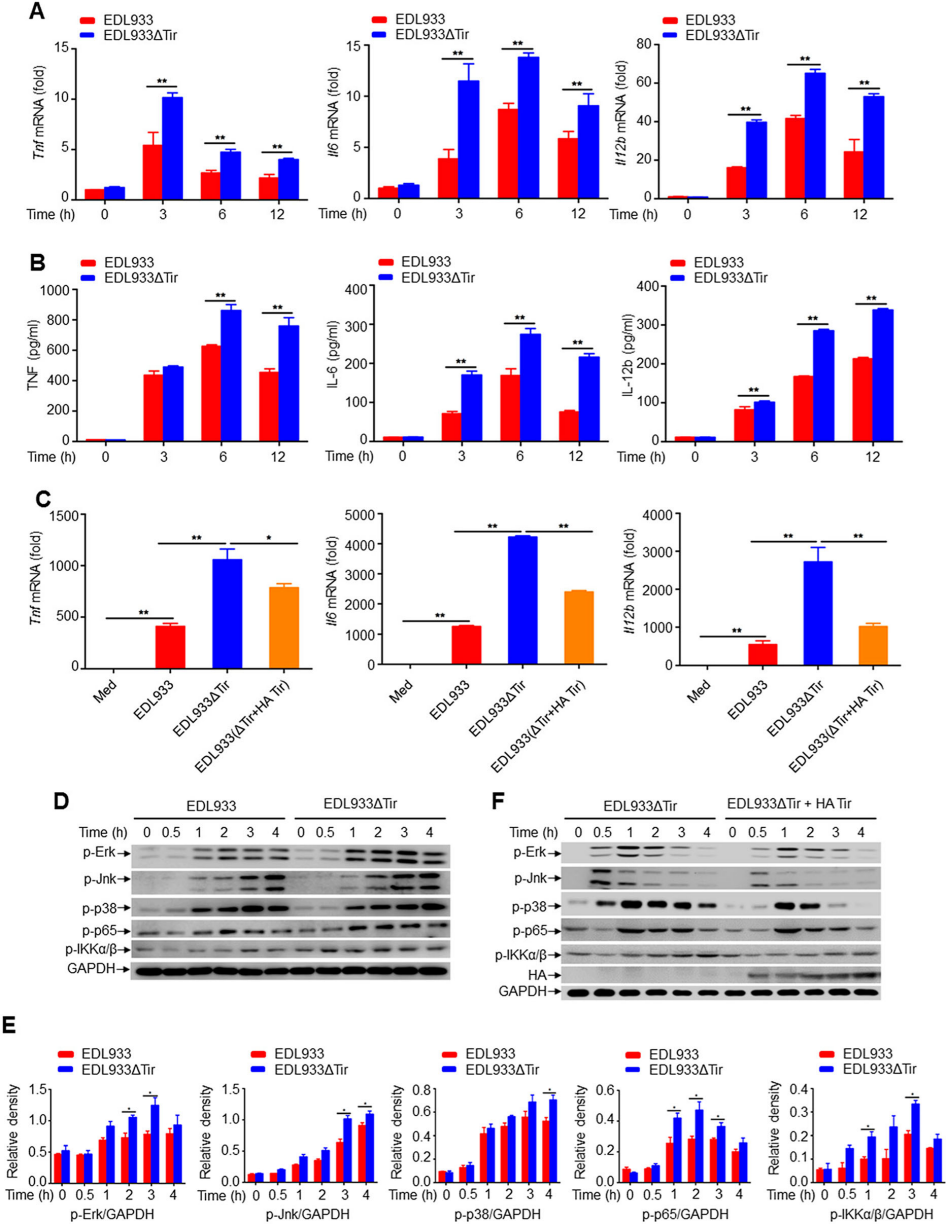
EHEC Tir inhibits cytokine production *in vitro.***a** Quantitative RT–PCR analysis of *Tnf*, *Il6,* and *Il12b* mRNAs in mouse primary peritoneal macrophages infected with EDL933 or EDL933ΔTir for the indicated times. **b** ELISA analysis of TNF, IL-6, and IL-12b in the supernatants of mouse primary peritoneal macrophages infected with EDL933 or EDL933ΔTir for the indicated times. **c** Quantitative RT–PCR analysis of *Tnf*, *Il6,* and *Il12b* mRNAs in mouse primary peritoneal macrophages infected with medium, EDL933, EDL933ΔTir or EDL933(ΔTir + HA-Tir). **d** Immunoassay of the indicated proteins in mouse primary peritoneal macrophages infected with EDL933 or EDL933ΔTir. **e** Image J analysis of the greyscale value of western blot bands in panel **d**, *n* = 3. **f** Immunoassay of lysates of or mouse primary peritoneal macrophages infected with EDL933ΔTir or EDL933(ΔTir + HA-Tir). Data are representative of at least three independent experiments (mean ± SEM in **a–c**). *p < 0.05 and **p < 0.01 (Student′s *t*-test).

To identify whether EHEC Tir is involved in bacteria-induced innate immune responses and proinflammatory cytokine production *in vitro*, we performed qPCR experiments to investigate the dynamic expression of proinflammatory cytokines in mouse primary peritoneal macrophages and RAW264.7 cells in response to the EDL933 or EDL933ΔTir strains. We found that the EDL933ΔTir strain induced more *Tnf, Il6,* and *Il12b* than the EDL933 strain in mouse primary peritoneal macrophages (Figure 2a) and RAW264.7 cells (Supplemental Figure 2a). The viability of RAW264.7 cells infected with EDL933 or EDL933ΔTir were the same (Supplemental Figure 2b). In addition, enzyme-linked immunosorbent assays (ELISA) showed that EDL933-induced secretion of TNF, IL-6, and IL-12b was also lower than that of EDL933ΔTir in the supernatant of mouse primary peritoneal macrophages (Figure 2b). These results suggest that the EHEC Tir protein inhibits cytokine production *in vitro*. To confirm whether EDL933 Tir inhibits cytokine production specifically, we infected mouse primary peritoneal macrophages with EDL933, EDL933ΔTir or EDL933(ΔTir + HA-Tir) (an EDL933ΔTir strain transformed with a HA-tagged Tir encoded by the vector pK184) strains to detect the production of *Tnf*, *Il6* and *Il12b* by qPCR (Figure 2c). We found that EDL933(ΔTir + HA-Tir) induced lower levels of proinflammatory cytokines than EDL933ΔTir, and both groups had similar levels of cell viability and phagocytic function (Supplemental Figure 2c-d). These results suggest that EHEC Tir specifically inhibits EHEC-triggered induction of proinflammatory cytokines.

We next detected whether EDL933 Tir modulated the activation of the MAP kinase and NF-κB signalling pathways, which regulate downstream proinflammatory cytokine production. Consistent with the cytokine results, EDL933ΔTir induced higher phosphorylation of Erk, Jnk, p38 MAPK, NF-κB p65, and IKKα/β than EDL933 in mouse primary peritoneal macrophages (Figure 2d-e), which was inhibited by the EDL933(ΔTir + HA-Tir) strain (Figure 2f) when the Tir protein was inserted into the host cell and phosphorylated (Supplemental Figure 3a-b). RNA sequencing results showed that some of the EDL933ΔTir-induced genes such as *Ror1*, *Prkdc,* and *Mras* were associated with inflammation (Supplemental Figure 3c). These results suggested that Tir might be an important protein in suppression of EHEC-induced cytokine production.

### EHEC Tir recruits phosphatase SHP-1

**Figure 3.**
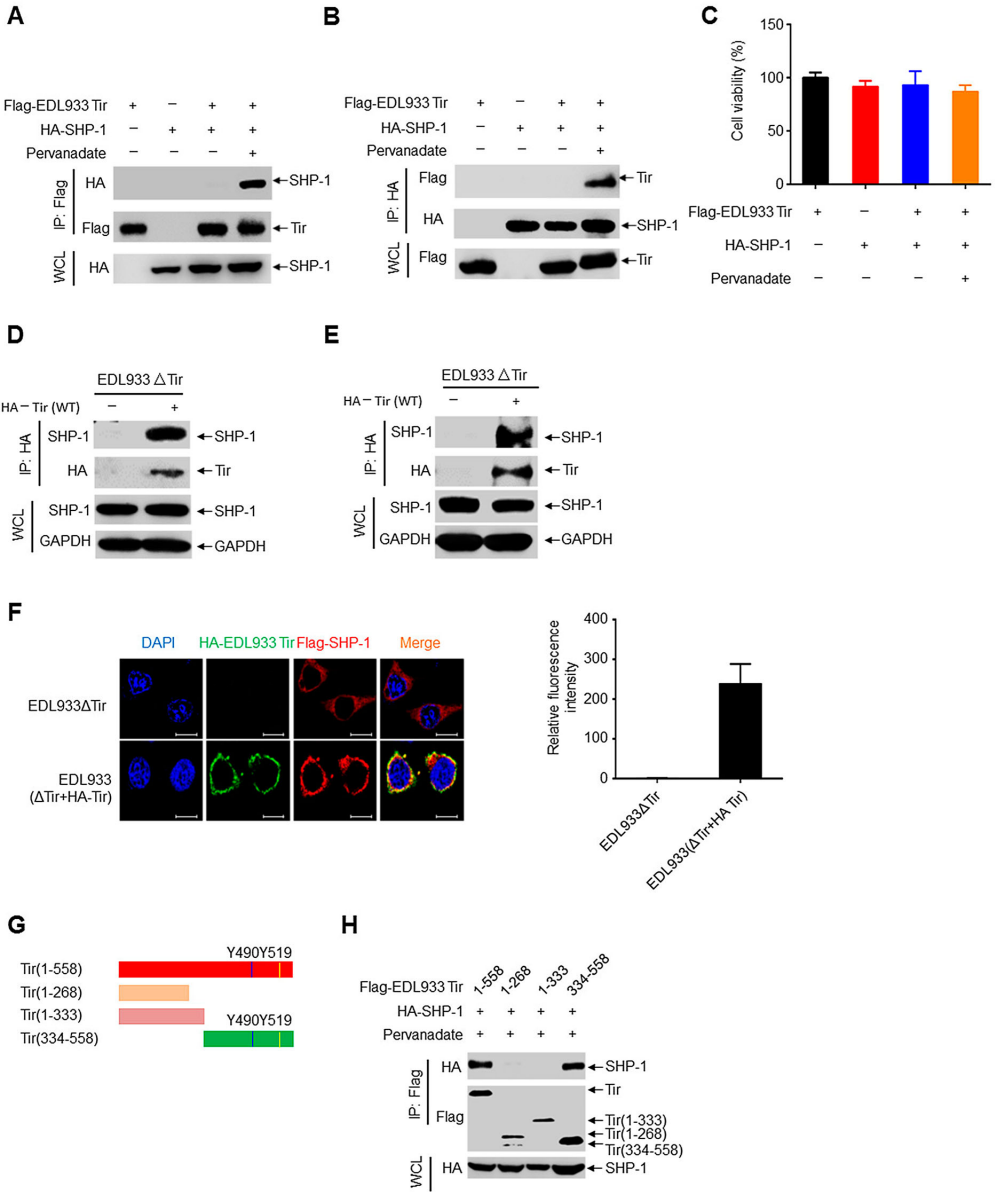
EHEC Tir recruits phosphatase SHP-1. **a-b** Immunoprecipitation (IP) and immunoblot (IB) analysis of Flag-EDL933 Tir and HA-SHP-1 in HEK293 T cells untreated or treated with pervanadate, probed with antibody to Flag or HA (left margin). **c** CCK-8 analysis of cell viability in HEK293 T cells treated as in **a** and **b**. **d-e** Immunoassay of RAW 264.7 cells (**d**) or mouse primary peritoneal macrophages (**e**) infected with EDL933ΔTir (−) or EDL933(ΔTir + HA-Tir) (+). **f** Immunofluorescence microscopy of HeLa cells transfected with Flag-SHP-1 and infected with EDL933ΔTir (top row) or EDL933(ΔTir + HA-Tir) (bottom row), stained with antibody to HA (green), Flag (red) and the DNA-binding dye DAPI (blue). Scale bars, 10 μm. Relative fluorescence intensity of Flag-SHP-1 was calculated with Image J from three different areas, each of which contains three cells (right graph). *n* = 3. **g** EDL933 Tir deletion mutants. **h** Co-immunoprecipitation assay of Flag-EDL933 Tir deletion mutants and HA-SHP-1 in HEK293 T cells. Data are representative of at least three independent experiments.

Using bioinformatics methods, we unexpectedly found that EHEC Tir contains two ITIMs. ITIMs can interact with SHP-1 upon tyrosine phosphorylation [33], which inhibits intracellular calcium mobilization and subsequent cell activation [34]. Co-immunoprecipitation experiments showed that EDL933 Tir interacted with SHP-1 in the presence of the tyrosine phosphatase inhibitor pervanadate (Figure 3a-b), while both groups had similar levels of cell viability (Figure 3c). Similarly, EDL933 Tir also immunoprecipitated with SHP-2 (Supplemental Figure 4a-b). However, EDL933 Tir did not interact with the SHP-1 (C453S) mutant, indicating that the interaction is dependent on SHP-1 phosphatase activity (Supplemental Figure 4c). Furthermore, we infected RAW264.7 cells or mouse primary peritoneal macrophages with EDL933ΔTir or EDL933(ΔTir + HA-Tir) to determine whether EDL933 Tir interacted with endogenous SHP-1. The results showed that Tir interacted with endogenous SHP-1 in RAW264.7 cells (Figure 3d) and mouse primary peritoneal macrophages (Figure 3e). Next, we determined the intracellular co-localization of EHEC Tir and SHP-1 by immunofluorescent confocal microscopy. HeLa cells were transfected with Flag-SHP-1 and then infected with EDL933ΔTir or EDL933(ΔTir + HA-Tir). The results showed that HA-Tir (green) colocalized with Flag-SHP-1 (red) (Figure 3f). To detect whether EHEC Tir and SHP-1 bind each other directly, we used competent TKB1 *E. coli* cells (encoding a tyrosine kinase to induce expression of tyrosine-phosphorylated proteins) to express the recombinant GST fusion EHEC Tir and incubated it with purified recombinant histidine-tagged SHP-1 or endogenous SHP-1 from RAW264.7 cells. The results showed that EDL933 Tir proteins interacted directly with His-SHP-1, the endogenous SHP-1 from RAW264.7 cells, and mouse primary peritoneal macrophages (Supplemental Figure 4d-f). To identify the binding region of EHEC Tir and SHP-1, we constructed a series of deletion mutants of EDL933 Tir and SHP-1 (Figure 3 g, supplemental 4 g). The results showed that SHP-1 bound only to amino acid residues 334–558 of EDL933 Tir [containing the two ITIMs (Tyr490 and Tyr519)] but not to amino acid residues 1–333, indicating that ITIMs play an important role in the EDL933 Tir–SHP-1 interaction (Figure 3 h). Hence, the SHP-1-binding domain of EDL933 Tir may lie in residues 334–558. Similarly, EDL933 Tir bound to amino acid residues 107–595 or 221–595, but not 1–242 of SHP-1 (Supplemental Figure 4 h), suggesting that the C-terminus of SHP-1 is essential for the binding of Tir. Taken together, these results suggest that EHEC Tir interacts with tyrosine phosphatase SHP-1.

### SHP-1 inhibits TAK1 phosphorylation and ubiquitination

**Figure 4.**
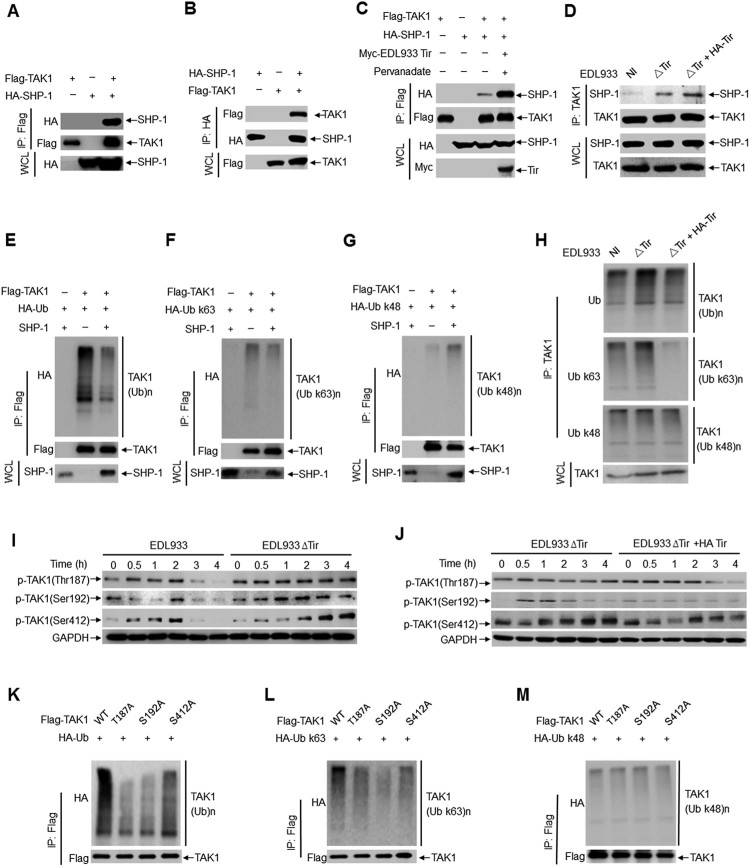
SHP-1 inhibits TAK1 phosphorylation and ubiquitination. **a-c** Co-immunoprecipitation assay of Flag-TAK1, HA-SHP-1 or Myc-EDL933 Tir expressed in HEK293 T cells. Immunoassay of mouse primary peritoneal macrophages uninfected (NI) or infected with EDL933ΔTir or EDL933(ΔTir + HA-Tir). **d** Immunoassay of mouse primary peritoneal macrophages uninfected (NI) or infected with EDL933ΔTir or EDL933(ΔTir + HA-Tir). **e-g** Immunoassay of HEK293 T cells expressing various vectors (above lanes). **h-j** Immunoassay of mouse primary peritoneal macrophages infected with indicated strains (above lanes). **k-m** Immunoassay of HEK293 T cells expressing TAK1 point mutants (T187A, S192A, S412A) and ubiquitin (total, K48 or K63 ubiquitin). Data are representative of at least three independent experiments.

TAK1 is widely accepted as a key regulator in proinflammatory cytokine signalling, which can be activated by TNF, interleukin-1 (IL-1), and toll-like receptor (TLR) ligands [35,36]. LPS from Gram-negative bacteria stimulates the TLR4-MyD88-TRAF6-TAK1 axis and leads to the activation of the MAPK and NF-κB pathways, which cooperatively regulate the transcription of genes involved in the inflammatory immune response [37–39]. Phosphorylation and ubiquitination of TAK1 regulate this pathway. Using lysates of HEK293 T cells expressing Flag-tagged TAK1 and HA-tagged SHP-1, we determined that SHP-1 immunoprecipitated with TAK1 in a co-immunoprecipitation assay (Figure 4 a-b). An *in vitro* GST pull-down assay showed that TAK1 interacted directly with purified recombinant His-SHP-1, endogenous SHP-1 from RAW264.7 cells and mouse primary peritoneal macrophages (Supplemental Figure 5a-c). However, TAK1 did not interact with the SHP-1 (C453S) mutant, indicating that the interaction is dependent on SHP-1 phosphatase activity (Supplemental Figure 5d). Similarly, SHP-2 co-immunoprecipitated with TAK1 (Supplemental Figure 5e). Furthermore, overexpression of EDL933 Tir in HEK293 T cells significantly enhanced the association of TAK1 and SHP-1 (Figure 4c) but did not enhance the association of TRAF6 and SHP-1 (Supplemental Figure 5f). The endogenous interaction between SHP-1 and TAK1 was enhanced in mouse primary peritoneal macrophage cells infected with EDL933(ΔTir + HA-Tir) (Figure 4d).

TAK1 depends on its phosphorylation and polyubiquitination to modulate downstream signalling. As EDL933 Tir enhanced the association of TAK1 and SHP-1, we examined the effect of Tir and SHP-1 on the phosphorylation and polyubiquitination of TAK1. The results showed that overexpression of SHP-1 resulted in less total ubiquitination (Figure 4e) and K63-linked polyubiquitination of TAK1 (Figure 4f) but had no effect on the K48-linked polyubiquitination (Figure 4 g) in HEK293 T cells. Infection of mouse primary peritoneal macrophages with EDL933(ΔTir + HA-Tir) elicited less total ubiquitination and K63-linked polyubiquitination of TAK1 than infection with EDL933ΔTir but did not affect K48-linked polyubiquitination (Figure 4 h).

Several threonine and serine residues in TAK1, including Thr-187, Ser-192, and Ser-412, are phosphorylated upon cytokine stimulation to induce TAK1 activation [15,40,41]. By immunoblot analysis, we found that the levels of phosphorylated TAK1 (*p*-TAK1 Thr187, Ser192, Ser412) were significantly increased in mouse primary peritoneal macrophages (Figure 4i) and RAW264.7 cells (Supplemental Figure 5 g) infected with EDL933ΔTir compared to those infected with EDL933. Furthermore, the levels of phosphorylated TAK1 were significantly decreased in mouse primary peritoneal macrophages (Figure 4j) infected with EDL933(ΔTir + HA-Tir) compared to those infected with EDL933ΔTir.

As EHEC Tir promotes SHP-1 to interact with TAK1 and inhibit TAK1 phosphorylation and K63-linked polyubiquitination, we wanted to determine whether SHP-1 inhibits TAK1 polyubiquitination by regulating its own phosphorylation. To test this hypothesis, we constructed three TAK1 phosphorylation point mutants (T187A, S192A, S412A). The results showed that TAK1(T187A) and TAK1(S192A) decreased total ubiquitination (Figure 4k) and K63-linked polyubiquitination (Figure 4 l) but not K48-linked polyubiquitination (Figure 4 m) of TAK1. Thus, these results suggest that phosphorylation of TAK1 promotes K63-linked polyubiquitination. In other words, Tir promotes SHP-1 to inhibit K63-linked polyubiquitination of TAK1 by inhibiting TAK1 phosphorylation. However, the mechanism by which TAK1 phosphorylation regulates its own ubiquitination needs to be further elucidated.

### EHEC Tir–SHP-1 interaction and cytokine inhibition depend on ITIM tyrosine phosphorylation

**Figure 5.**
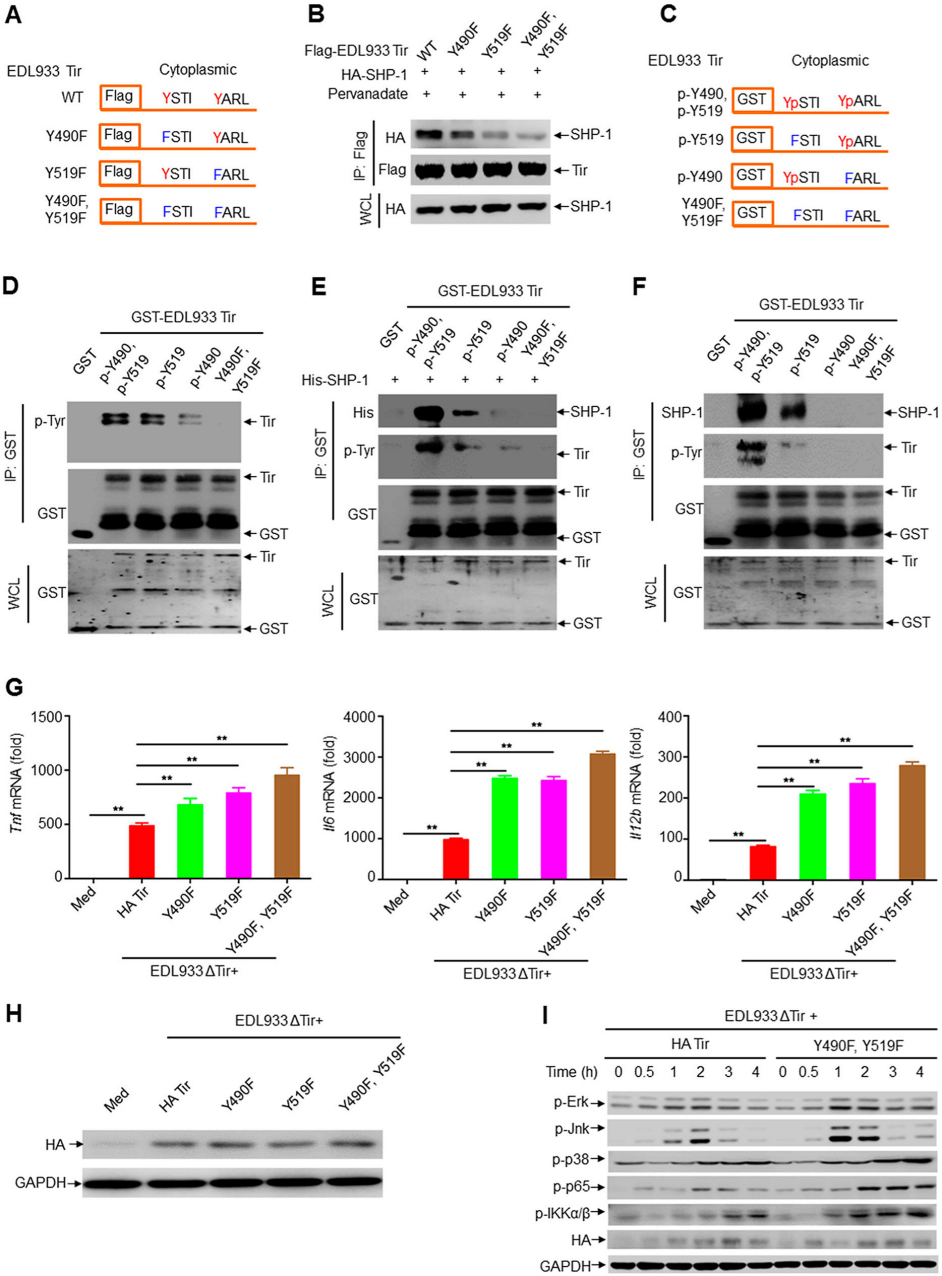
EHEC Tir–SHP-1 interaction and cytokine inhibition are dependent on ITIM tyrosine phosphorylation. **a** EDL933 Tir ITIM mutants. Red indicates tyrosine in the wild-type (WT) site that can be tyrosine-phosphorylated; blue indicates phenylalanine in the mutant residue that cannot be tyrosine-phosphorylated. **b** Immunoassay of lysates of HEK293 T cells expressing SHP-1 and wild-type EDL933 Tir or its ITIM mutants in A (top). **c** GST fusion mutants of EDL933 Tir. *p*-Y and Yp indicate phosphorylated tyrosine. Tyrosine phosphorylation of GST-Tir (second lane) or its ITIM mutants purified from TKB1 that expresses tyrosine kinase. **d-f** GST precipitation assay of GST-EDL933 Tir or its ITIM mutants (**d**) with purified His-SHP-1 (**e**) or endogenous SHP-1 from primary macrophage (**f**). **g-h** Quantitative RT–PCR analysis of *Tnf*, *Il6* or *Il12b* mRNAs (**g**) and immunoassay the protein of HA-Tir (**h**) in mouse primary peritoneal macrophages left uninfected (Med) or infected for 6 h with EDL933(ΔTir + HA-Tir), EDL933(ΔTir + Y490F), EDL933(ΔTir + Y519F) or EDL933(ΔTir + Y490F, Y519F). **i** Immunoassay of lysates of mouse primary peritoneal macrophages infected with EDL933(ΔTir + HA-Tir) or EDL933(ΔTir + Y490F, Y519F) for the indicated times. Data are representative of at least three independent experiments (mean ± SEM in **g**). **p < 0.01 (Student’s *t*-test).

Host cellular ITIM-containing proteins must be phosphorylated on both ITIMs to interact with SHP-1 [42]. We constructed EDL933 Tir mutants containing two sites of ITIM in which either one or both ITIMs were replaced with phenylalanine to determine whether Tir and SHP-1 require tyrosine phosphorylation in ITIMs (Figure 5a). The results showed that the Y490F and Y519F mutants resulted in less co-immunoprecipitation with SHP-1 than WT Tir (Figure 5b). Similarly, we observed that EDL933 Tir mutants had a lower interaction with SHP-2 (Supplemental Figure 6a-b). A GST pull-down assay showed that tyrosine phosphorylation of GST-Tir disappeared when the tyrosine residues in ITIMs were transformed to phenylalanine (Figure 5c). Consistently, only EDL933 Tir proteins with fully phosphorylated tyrosine (Figure 5d) in the ITIMs associated with purified recombinant histidine-tagged SHP-1 (Figure 5e) or endogenous SHP-1 (Figure 5f) from mouse primary peritoneal macrophages, while the tyrosine residue mutants had a lower association. These results suggest that EHEC Tir requires tyrosine phosphorylation in ITIMs to interact with SHP-1.

We constructed the bacterial strains EDL933(ΔTir + HA-Tir), EDL933(ΔTir + Y490F), EDL933(ΔTir + Y519F), and EDL933(ΔTir + Y490F, Y519F) (Figure 5 h). The infection results showed that EDL933(ΔTir + Y490F) and EDL933(ΔTir + Y519F) induced much higher production of *Tnf*, *Il6* and *Il12b* than the EDL933(ΔTir + HA-Tir) strain in mouse primary peritoneal macrophages, and EDL933(ΔTir + Y490F, Y519F) induced even higher cytokine production (Figure 5 g). Moreover, we obtained similar results in RAW264.7 cells (Supplemental Figure 6c). In addition, EDL933(ΔTir + Y490F), EDL933(ΔTir + Y519F), and EDL933(ΔTir + Y490F, Y519F) induced higher secretion of TNF, IL-6, and IL-12b than EDL933(ΔTir + HA-Tir) in the primary macrophage supernatants (Supplemental Figure 6d). Consistently, EDL933(ΔTir + Y490F, Y519F) induced much higher phosphorylation of Erk, Jnk, p38 MAPK, NF-κB p65, and IKKα/β than EDL933(ΔTir + HA-Tir) in mouse primary peritoneal macrophages (Figure 5i). Thus, the Tir–SHP-1 interaction and cytokine inhibition are dependent on ITIM tyrosine phosphorylation.

### ITIMs of EHEC Tir specifically inhibit intestinal immunity

**Figure 6.**
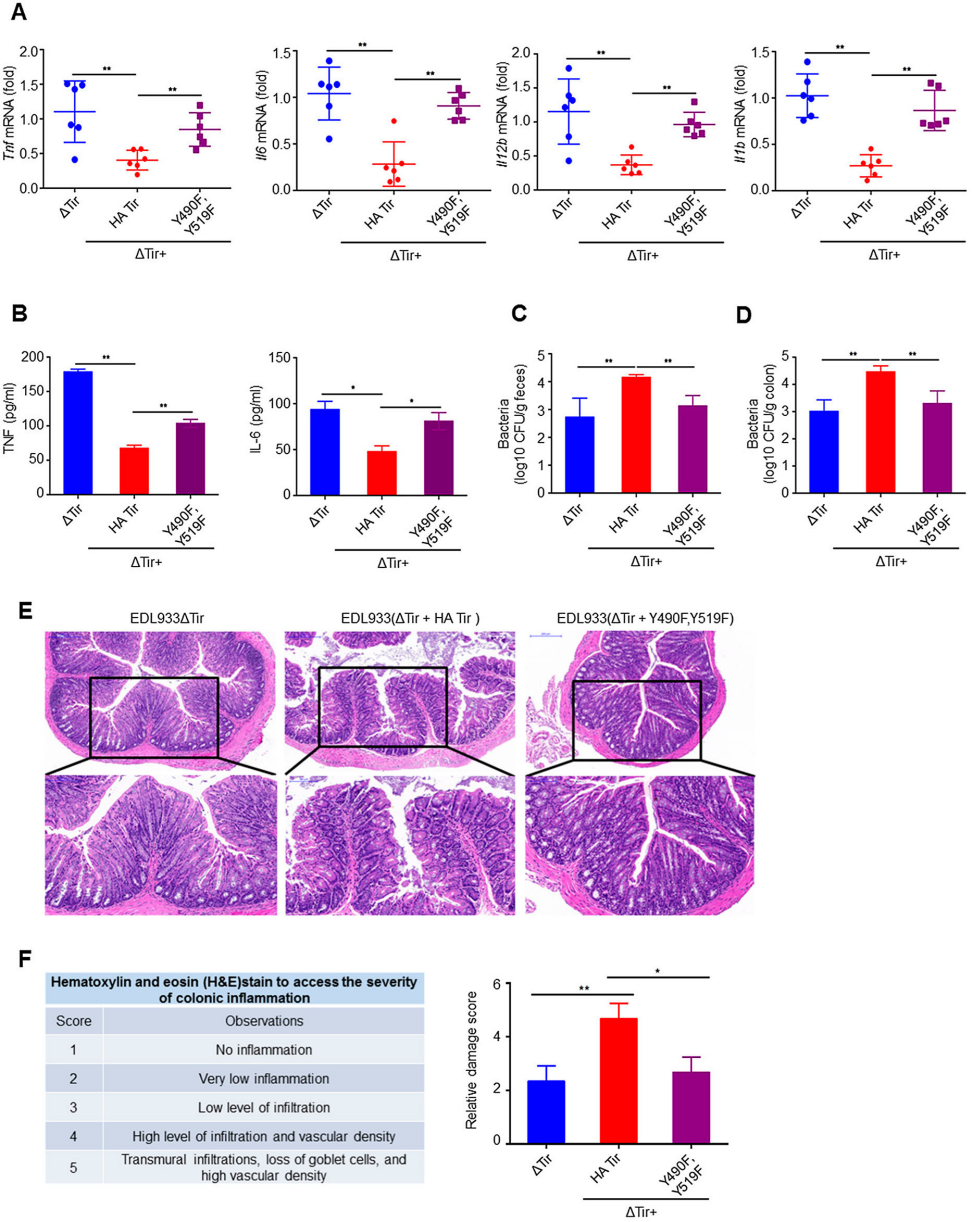
ITIMs in EHEC Tir specifically inhibit intestinal immunity. **a** Quantitative RT–PCR analysis of *Tnf*, *Il6*, *Il12b* or *Il1b* mRNAs in colon from 3-4-week-old mice (*n* = 6 per group) infected orally with 1 × 10^9^ CFU of EDL933ΔTir, EDL933(ΔTir + HA-Tir) or EDL933(ΔTir + Y490F, Y519F). **b** ELISA assay of TNF or IL-6 from mouse serum in **a**. **c-d** Bacterial load in the feces (**c**) or colon (**d**) of mice infected as in **a** at the indicated times. **e** Histopathology of colons of mice left uninfected (NI) or infected as in **a**. Outlined areas at top are enlarged below. Original magnification, ×10 (top) or ×20 (bottom). **f** Hematoxylin and eosin (H&E) stain to access the severity of colonic inflammation. Data are representative of at least two independent experiments (mean ± SEM in **a–d, f**). *p < 0.05 and **p < 0.01 (Student’s *t*-test).

With the infection mouse model above, we found that EDL933(ΔTir + HA-Tir) induced lower *Tnf, Il6, Il12b,* and *Il1b* than EDL933ΔTir, which was nearly reversed by EDL933(ΔTir + Y490F, Y519F) in the colons (Figure 6a) and spleens (Supplemental Figure 7a). In addition, EDL933(ΔTir + HA-Tir) inhibition of TNF and IL-6 secretion was rescued by EDL933(ΔTir + Y490F, Y519F) in the serum (Figure 6b). In contrast, the EDL933(ΔTir + Y490F, Y519F) CFUs were significantly less than those of EDL933(ΔTir + HA-Tir) in the feces (Figure 6c) and colon (Figure 6d), which was comparable to EDL933ΔTir. Conversely, mice infected with EDL933ΔTir or EDL933(ΔTir + Y490F, Y519F) had greater increases in body weight than EDL933(ΔTir + HA-Tir) infected mice (Supplemental Figure 7b). Histological examination of mice infected with EDL933(ΔTir + Y490F, Y519F) revealed less extensive neutrophil inflammatory infiltrates and structural disruption of the colonic epithelium than EDL933(ΔTir + HA-Tir) infected mice (Figure 6e-f). These results suggest that tyrosine phosphorylation of the ITIMs of Tir may have inhibited intestinal immunity during EDL933 infection.

### Inhibition of cytokine production by EHEC Tir is mediated by SHP-1

**Figure 7.**
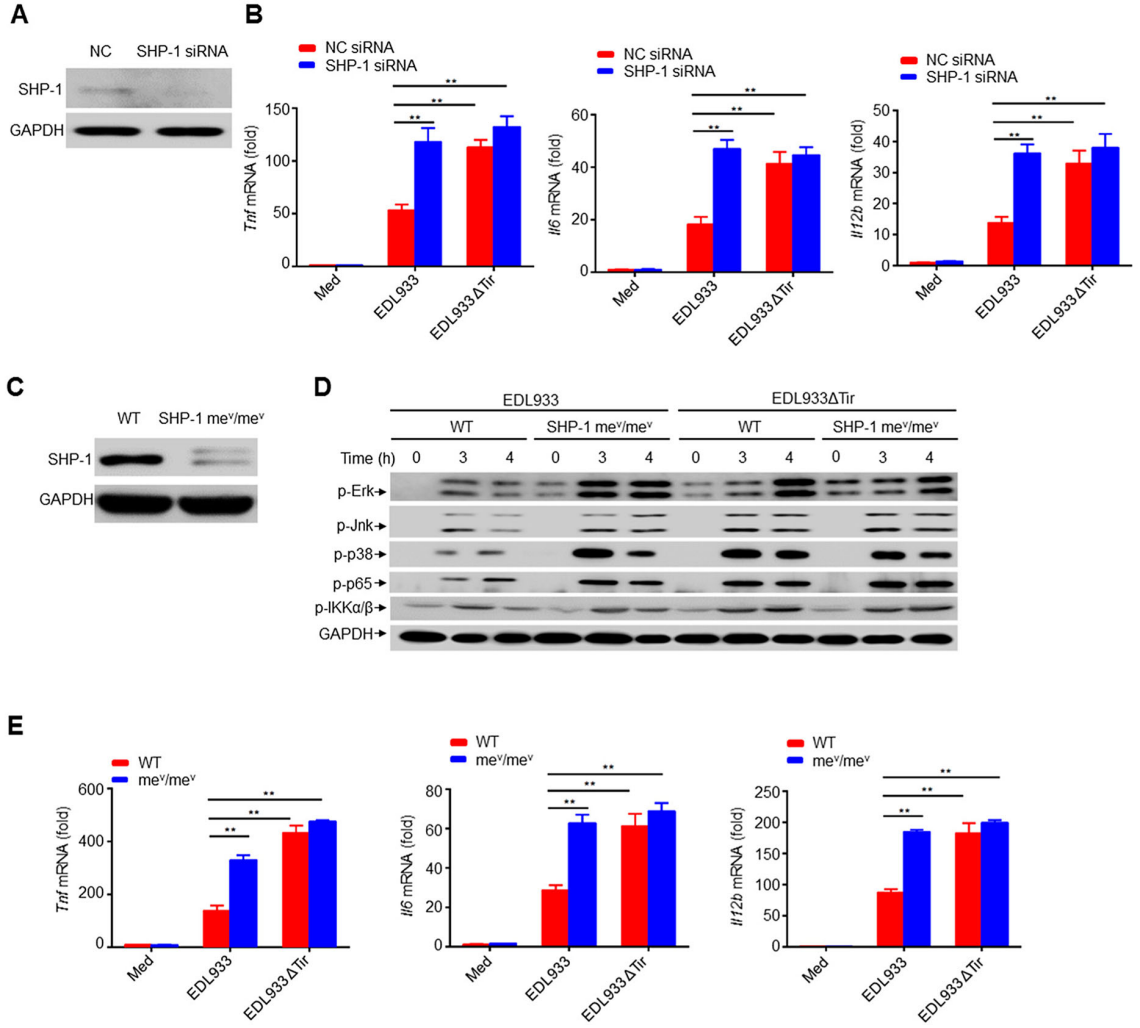
Inhibition of cytokine production by EHEC Tir is mediated by SHP-1. **a** Immunoassay of lysates of RAW264.7 cells transfected with negative control siRNA (NC) or SHP-1-specific siRNA. **b** Quantitative RT–PCR analysis of *Tnf*, *Il6* or *Il12b* mRNAs in RAW264.7 cells transfected with siRNA and then left uninfected (Med) or infected with EDL933 or EDL933ΔTir. **c** Expression of SHP-1 in mouse primary peritoneal macrophages from wild-type (WT) or *Ptpn6 m*^e-v/me-v^ (me^v^/me^v^) mice. **d** Immunoassay of lysates of WT or me^v^/me^v^ primary peritoneal macrophages infected with EDL933 or EDL933ΔTir for the indicated times. **e** Quantitative RT–PCR analysis of *Tnf*, *Il6* or *Il12b* mRNAs in WT or me^v^/me^v^ primary peritoneal macrophages infected for 6 h with EDL933 or EDL933ΔTir. Data are representative of at least three independent experiments (mean ± SEM in **b**, **e**). **p < 0.01 (Student’s *t*-test).

SHP-1-deficient mice have confirmed an integral role for SHP-1 in negatively regulating cell activation to modulate the immune response ^[^43,44]. To determine the role of SHP-1 in Tir-mediated inhibition of cytokine production, we transfected RAW264.7 cells with negative control small interfering RNA (siRNA) (NC) or SHP-1-specific siRNA by electroporation (Figure 7a). We infected cells with EDL933 or EDL933ΔTir and observed that the SHP-1 siRNA group infected with EDL933 induced much higher production of *Tnf*, *Il6*, *Il12b,* and *Il1b* than the NC group, whereas EDL933ΔTir induced relatively high cytokine production in both the NC and SHP-1 siRNA groups (Figure 7b, supplemental 8a).

We isolated mouse primary peritoneal macrophages from viable motheaten strain (*Ptpn6*^me-v^/^me-v^) mice, which have low expression of SHP-1 (Figure 7c), and infected mouse primary peritoneal macrophages with EDL933 or EDL933ΔTir, respectively. The SHP-1 me^v^/me^v^ group infected with EDL933 had significantly higher phosphorylation levels of Erk, Jnk, p38 MAPK, NF-κB p65, and IKKα/β, as well as significantly higher production of *Tnf*, *Il6*, *Il12b,* and *Il1b* than the WT group, whereas EDL933ΔTir induced relatively high phosphorylation of these kinases and cytokine production in both WT and SHP-1 me^v^/me^v^ cells (Figure 7d-e, supplemental 8b). Thus, SHP-1 could be a critical regulator in Tir-mediated inhibition of cytokine production.

## Discussion

The EHEC effector protein Tir, which is injected into the plasma membrane of host cells via bacterial T3SS and localized at sites of bacterial attachment [25] adopts a hairpin loop conformation and serves as a receptor for the bacterial surface adhesin intimin [23]. EHEC generates AE lesions to colonize the intestine, damage the epithelium and promote diarrheal illnesses [21,22]. These lesions are characterized by localized replacement of microvilli with organized filamentous (F-) actin ‘pedestals’ beneath intimately adherent bacteria [45,46]. The binding of intimin and the central extracellular domain of Tir promotes clustering of the N- and C-terminal cytoplasmic regions and initiates localized actin assembly beneath the plasma membrane of intestinal epithelial cells [26]. However, the regulatory role of EHEC Tir in immune cells remains unknown. By analyzing an EHEC infection model, we uncovered a pathway by which EHEC Tir inhibited immune responses by recruiting SHP-1 in an ITIM tyrosine phosphorylation-dependent manner. SHP-1 inhibits TAK1 activity to down-regulate signal transduction and subsequent cytokine production.

Innate immune responses are achieved by the activation of several pathogen-recognition receptors (PRPs), including TLRs, retinoic acid inducible gene I (RIG-I)-like receptors (RLRs) and nucleotide-binding oligomerization domain (NOD)-like receptors (NLRs). In mammals, PRPs utilize TAK1 to activate NF-κB through myeloid differentiation factor 88 (MyD88) in macrophages [47]. Although EHEC Tir is essential for the assembly of F-actin pedestals [48], relatively little is known about how Tir regulates innate inflammatory responses. The data obtained in our study demonstrate that Tir inhibits EHEC-induced expression of the proinflammatory cytokines TNF and IL-6 *in vitro* and *in vivo*. Our observation of lower bacterial burdens in mice infected with EDL933 ΔTir may be due to the higher expression of TNF and IL-6. Thus, in addition to its role in pedestal formation, EHEC Tir also contributes to the pathogenicity of EHEC by inhibiting host proinflammatory responses.

TAK1 is widely accepted as a key regulator in proinflammatory cytokine signalling, which can be activated by TNF, IL-1, and TLR ligands [35,36]. Several threonine and serine residues in TAK1, including Thr-187, Ser-192, and Ser-412, are phosphorylated upon cytokine stimulation [15,40,41]. We found that EHEC Tir inhibited TAK1 phosphorylation at Thr-187, Ser-192, and Ser-412. EHEC Tir enhanced the interaction between TAK1 and SHP-1, which then inhibited K63-linked polyubiquitination. These data indicate that EHEC Tir may inhibit TAK1 activation through SHP-1. However, little is known about the relationship between the phosphorylation and ubiquitination of TAK1. We found that two TAK1 point mutants, TAK1(T187A) and TAK1(S192A), decreased total and K63-linked polyubiquitination of TAK1 and that EHEC Tir down-regulated TAK1 phosphorylation at Thr-187 and Ser-192. Thus, we propose that Tir might promote SHP-1 to inhibit K63-linked TAK1 polyubiquitination by inhibiting its phosphorylation. However, the mechanism by which TAK1 phosphorylation regulates its own ubiquitination must be further elucidated.

In summary, the results of this study show that EHEC Tir interacted with the host cellular tyrosine phosphatase SHP-1 in an ITIM phosphorylation-dependent manner to inhibit proinflammatory cytokines and intestinal immunity. The association of Tir with SHP-1 facilitated the recruitment of SHP-1 to TAK1 and inhibited phosphorylation of TAK1, which is negatively related to TAK1, MAPK, and NF-κB activation. Moreover, Tir may promote SHP-1 to inhibit K63-linked TAK1 polyubiquitination by mechanically inhibiting its phosphorylation (Fig. S9). Taken together, these results not only provide a new molecular basis for the immune evasion mechanisms of pathogens, but also supply important theoretical evidence for the development of clinical strategies for the treatment of infectious diseases.

## Supplementary Material

Supplemental Material
