# Supplementary material for: Enterohemorrhagic Escherichia coli Tir inhibits TAK1 activation and mediates immune evasion
Source: Emerg Microbes Infect. 2019 May 25;8(1):734–48. doi: 10.1080/22221751.2019.1620589 (PMC6542180; doi:10.1080/22221751.2019.1620589)

**Enterohemorrhagic *Escherichia coli* Tir** **inhibits TAK1 activation and mediates immune evasion**

Ruixue Zhou^1,6^([zhouruixue125@163.com](mailto:zhouruixue125@163.com)), Zijuan Chen^1,6^([purplen_czj@126.com](mailto:purplen_czj@126.com)), Doudou Hao^1^([doudouhao@hotmail.com](mailto:doudouhao@hotmail.com)), Yu Wang^2^([xiaoyu027111@163.com](mailto:xiaoyu027111@163.com)), Yihua Zhang^1^([12307120273@fudan.edu.cn](mailto:12307120273@fudan.edu.cn)), Xianfu Yi^3^([yixfbio@gmail.com](mailto:yixfbio@gmail.com)), Liang-Dong Lyu^1^([ld.lyu@fudan.edu.cn](mailto:ld.lyu@fudan.edu.cn)), Haipeng Liu^4^([haipengliu2013@163.com](mailto:haipengliu2013@163.com)), Quanming Zou^2^([qmzou2007@163.com](mailto:qmzou2007@163.com)), Yiwei Chu^1^([ywchu@shmu.edu.cn](mailto:ywchu@shmu.edu.cn)), Baoxue Ge^4,5^([gebaoxue@sibs.ac.cn](mailto:gebaoxue@sibs.ac.cn)) & Dapeng Yan^1,*^ ([dapengyan@fudan.edu.cn](mailto:dapengyan@fudan.edu.cn))

1Department of Immunology, School of Basic Medical Sciences & Shanghai Public Health Clinical Center, Key Laboratory of Medical Molecular Virology of MOE/MOH, Fudan University, Shanghai 200032, China

2Department of Microbiology and Biochemical Pharmacy, National Engineering Research Centre of Immunological Products, College of Pharmacy, Army Medical University, Chongqing 400038, China

3School of Biomedical Engineering, Tianjin Medical University, Tianjin 300070, China

4Shanghai Key Laboratory of Tuberculosis, Shanghai Pulmonary Hospital, Tongji University School of Medicine, Shanghai 200092, China

5Department of Microbiology and Immunology, Tongji University School of Medicine, Shanghai 200092, China

6These authors contributed equally

Correspondence should be addressed to D.Y. ([dapengyan@fudan.edu.cn](mailto:dapengyan@fudan.edu.cn)), telephone: 86-21-54237692

Supporting Online Materials

Supplementary Figures 1-9

**Fig. supplemental 1 Tir inhibits immune responses to EHEC *in vivo*. a** qPCR analysis of *Tnf*, *Il6*, *Il12b*, *Il1b* and *Il10* mRNA in spleen from 3-4-week-old mice (n = 6 per group) infected orally with 1 × 10^9^ CFU EDL933 or EDL933∆Tir and sacrified at fourteenth day. **b** The body increasing weights of mice (mean ± SEM). ***P* < 0.01 (Student′s *t*-test). **c** F4/80, CD3, CD19-specific immunohistochemistry in the colon of EDL933 or EDL933∆Tir-infected mice (n = 3 mice per group). Scale bars, 100 μm. Relative intensity of F4/80, CD3 and CD19 was calculated with Image J from three different areas, each of which contains three samples (right graph). n = 3.

**Fig. supplemental 2 EHEC Tir specifically inhibits cytokine production. a** qPCR analysis of *Tnf*, *Il6* and *Il12b* mRNA in RAW264.7 cells infected for 0-12 h (horizontal axes) with EDL933 or EDL933ΔTir. Data are representative of at least three independent experiments (mean ± SEM). **b** CCK-8 analysis of cell viability in RAW264.7 cells infected with EDL933 or EDL933ΔTir for the indicated times. **c** CCK-8 analysis of cell viability in mouse primary peritoneal macrophages infected with medium, EDL933, EDL933ΔTir or EDL933(ΔTir + HA Tir). **d** Giemsa staining to detect the phagocytic role of mouse peritoneal macrophages infected with medium, EDL933, EDL933ΔTir or EDL933(ΔTir + HA Tir). ***P* < 0.01 (Student′s *t*-test).

**Fig. supplemental 3 Effect of Tir in EHEC-infected primary macrophages. a** EDL933∆Tir or EDL933(∆Tir+HA-Tir) bacteria were lysed by ultra-sonication, and the cell lysate was immunoprecipitated using an HA antibody and analyzed with p-Tyr or HA antibody. **b** IP and IB analysis of mouse primary peritoneal macrophages infected with the EDL933∆Tir or EDL933(∆Tir+HA-Tir) strains for the indicated times. **c** RNA-seq analysis of gene expression in mouse primary peritoneal macrophages infected with EDL933 or EDL933ΔTir for 6 h. Heatmap of total mRNA expression in mouse primary peritoneal macrophages infected with EDL933 or EDL933ΔTir for 6 h. Forecast network model of proteins in mouse primary peritoneal macrophages interacting with each other. Data are representative of at least two independent experiments.

**Fig. supplemental 4 EHEC Tir interacts with SHP-1/2. a-b** Immunoprecipitation (IP) and immunoblot (IB) of the lysates of HEK293T cells transfected with Flag-tagged EDL933 Tir and HA-tagged SHP-2, and left untreated or treated with pervanadate, and probed with antibody against Flag or HA (left margin). **c** Co-immunoprecipitation assay of HA-SHP-1 or phosphatase-defective SHP-1 (HA-SHP-1 (C453S)) and Flag-EDL933 Tir in HEK293T cells. **d-f** GST-pull down assay of direct binding of EDL933 Tir with purified His-SHP-1 (**d**) or endogenous SHP-1 from RAW264.7 cells (**e**) or mouse primary peritoneal macrophages (**f**). **g** SHP-1 deletion mutants. **h** Co-immunoprecipitation assay of Flag-SHP-1 deletion mutants (top, from **g**) and HA-EDL933 Tir in HEK293T cells. Data are representative of at least three independent experiments.

**Fig. supplemental 5 EHEC Tir prevents phosphorylation of TAK1.** **a-c** GST-pull down assay of the interaction of TAK1 with purified histidine-tagged SHP-1 (His-SHP-1) (**a**) or endogenous SHP-1 from RAW264.7 cells (**b**) or mouse primary peritoneal macrophages (**c**). **d** Co-immunoprecipitation assay of Flag-TAK1, HA-SHP-1 and HA-SHP-1 (C453S) expressed in HEK293T cells. **e** Immunoassay of lysates from HEK293T cells expressing Flag-TAK1 and HA-SHP-2. **f** Co-immunoprecipitation assay of Flag-TRAF6, HA-SHP-1 and Myc-EHEC Tir expressed in HEK293T cells. **g** Immunoassay of RAW264.7 cells infected with EDL933 or EDL933ΔTir. Data are representative of at least three independent experiments.

**Fig. supplemental 6 EHEC Tir–SHP-2 interaction and cytokine inhibition is dependent on ITIM tyrosine phosphorylation.** **a** EDL933 Tir ITIM mutants. Red indicates the wild-type (WT) site that can be tyrosine-phosphorylated; blue indicates the mutant residue that cannot be tyrosine-phosphorylated (one-letter amino acid codes). **b** Immunoassay of lysates from HEK293T cells expressing SHP-2 and wild-type EDL933 Tir or the Tir ITIM mutants in **a** (top). **c** qPCR analysis of *Tnf*, *Il6* or *Il12b* mRNA in RAW264.7 cells treated with medium or infected for 6 h with EDL933(ΔTir + HA-Tir), EDL933(ΔTir + Y490F), EDL933(ΔTir + Y519F) or EDL933(ΔTir + Y490F, Y519F). **d** ELISA of TNF, IL-6 and IL-12 in the supernatants of uninfected mouse primary peritoneal macrophages left uninfected (Med) or infected for 6 h with EDL933(ΔTir + HA-Tir), EDL933(ΔTir + Y490F), EDL933(ΔTir + Y519F) or EDL933(ΔTir + Y490F, Y519F). Data are representative of at least three independent experiments (mean ± SEM). ***P* < 0.01 (Student′s *t*-test).

**Fig. supplemental 7 ITIMs of EHEC Tir specifically inhibit intestinal immunity. a** qPCR analysis of *Tnf*, *Il6*, *Il12b* or *Il1b* mRNAs in spleen from 3-4-week-old mice (n = 6 per group) infected orally with 1 × 10^9^ CFU EDL933ΔTir, EDL933(ΔTir + HA-Tir) or EDL933(ΔTir + Y490F, Y519F). **b** The body increasing weights of mice (mean ± SEM). ***P* < 0.01 (Student′s *t*-test).

**Fig. supplemental 8 Inhibition of *Il1b* by EHEC Tir is mediated by SHP-1. a** qPCR analysis of *Il1b* mRNA in RAW264.7 cells transfected for 48 h with siRNA and then left uninfected (Med) or infected with EDL933 or EDL933ΔTir. **b** Quantitative RT-PCR analysis of *Il1b* mRNA in wild-type or *Ptpn6*^me-v/me-v^ primary peritoneal macrophages and then left uninfected (Med) or infected for 6 h with EDL933 or EDL933ΔTir. Data are representative of at least three independent experiments (mean ± SEM). ***P* < 0.01 (Student′s *t*-test).

**Fig. supplemental 9 Diagram depicting the negative regulation of signal transduction by EHEC Tir through ITIM-SHP-1-TAK1 axis.**

**Table. supplemental 1 Primers as follow,**

| Flag-EDL933 Tir | Forward | GACGATGATGACGCCGGATCCATGCCTATTGGTAATCTTGGT |
| --- | --- | --- |
|  | Reverse | CCCTCTAGATGCATGCTCGAGTTAGACGAAACGATGGGATCC |
| Flag-EDL933 Tir Y490F | Forward | GGGAATACAGATTCTGTTGTA**TTT**AGCACCATTCAACATCCTCCC |
|  | Reverse | GGGAGGATGTTGAATGGTGCTAAATACAACAGAATCTGTATTCCC |
| Flag-EDL933 Tir Y519F | Forward | AGTGCGGGGATTCAAAGCACT**TTT**GCGCGTCTGGCGCTAAGTGGT |
|  | Reverse | ACCACTTAGCGCCAGACGCGCAAAAGTGCTTTGAATCCCCGCACT |
| HA-SHP-1 (C453S) | Forward | GGGCCCATCATCGTGCACTCCAGCGCCGGCATCGGCCGC |
|  | Reverse | GCGGCCGATGCCGGCGCTGGAGTGCACGATGATGGGCCC |
| *Tnf* | Forward | TTCTGTCTACTGAACTTCGGGGTGATCGGT |
|  | Reverse | GTATGAGATAGCAAATCGGCTGACGGTGTGGG |
| *II6* | Forward | TCCAGTTGCCTTCTTGGGAC |
|  | Reverse | GTGTAATTAAGCCTCCGACTTG |
| *II12b* | Forward | GAGCACTCCCCATTCCTACT |
|  | Reverse | CCCTCCTCTGTCTCCTTCAT |
| *Il1b* | Forward | CCTCGTGCTGTCGGACCCATA |
|  | Reverse | CAGGCTTGTGCTCTGCTTGTGA |
| *Gapdh* | Forward | CCCACTAACATCAAATGGGG |
|  | Reverse | CCTTCCACAATGCCAAAGTT |


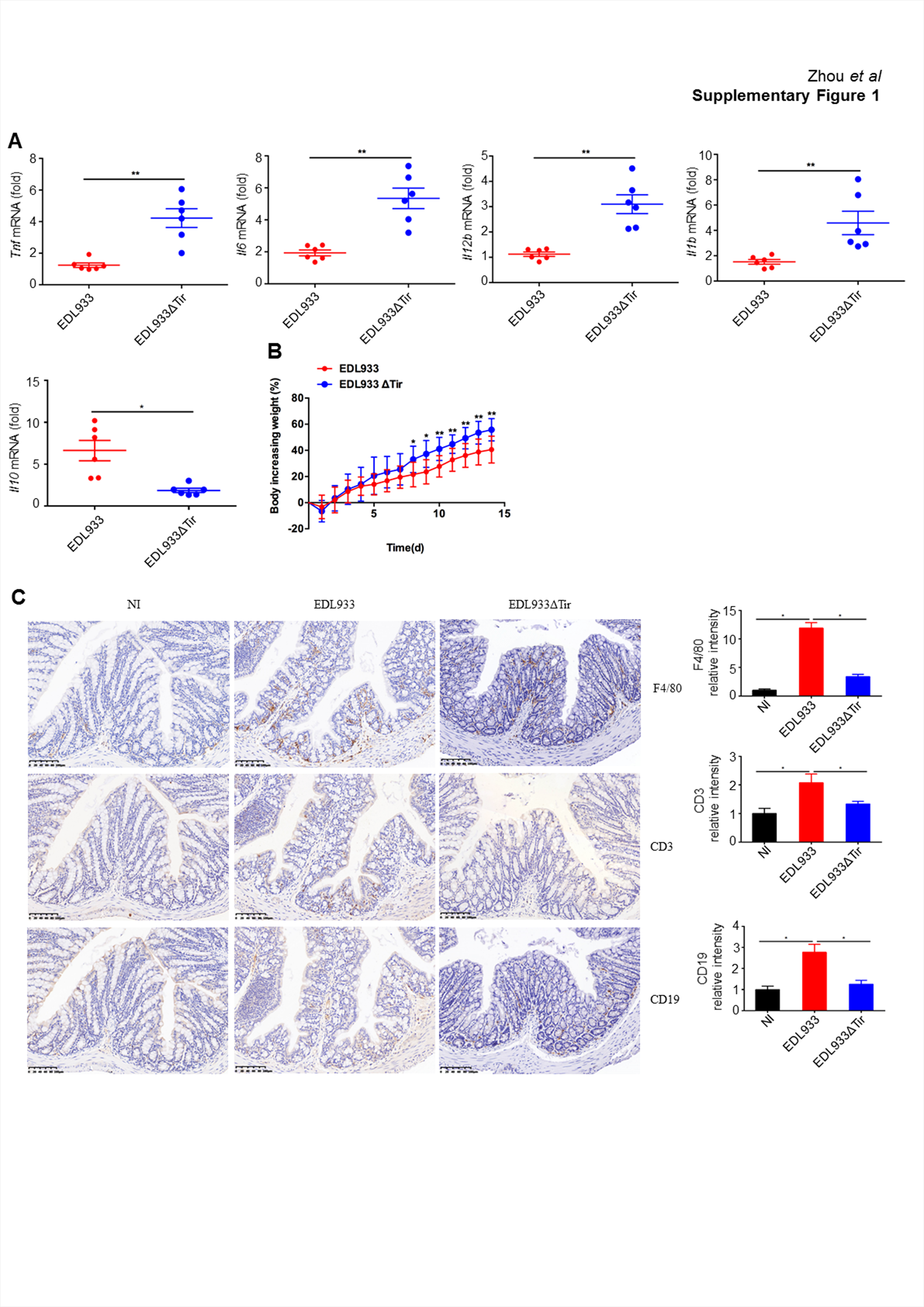


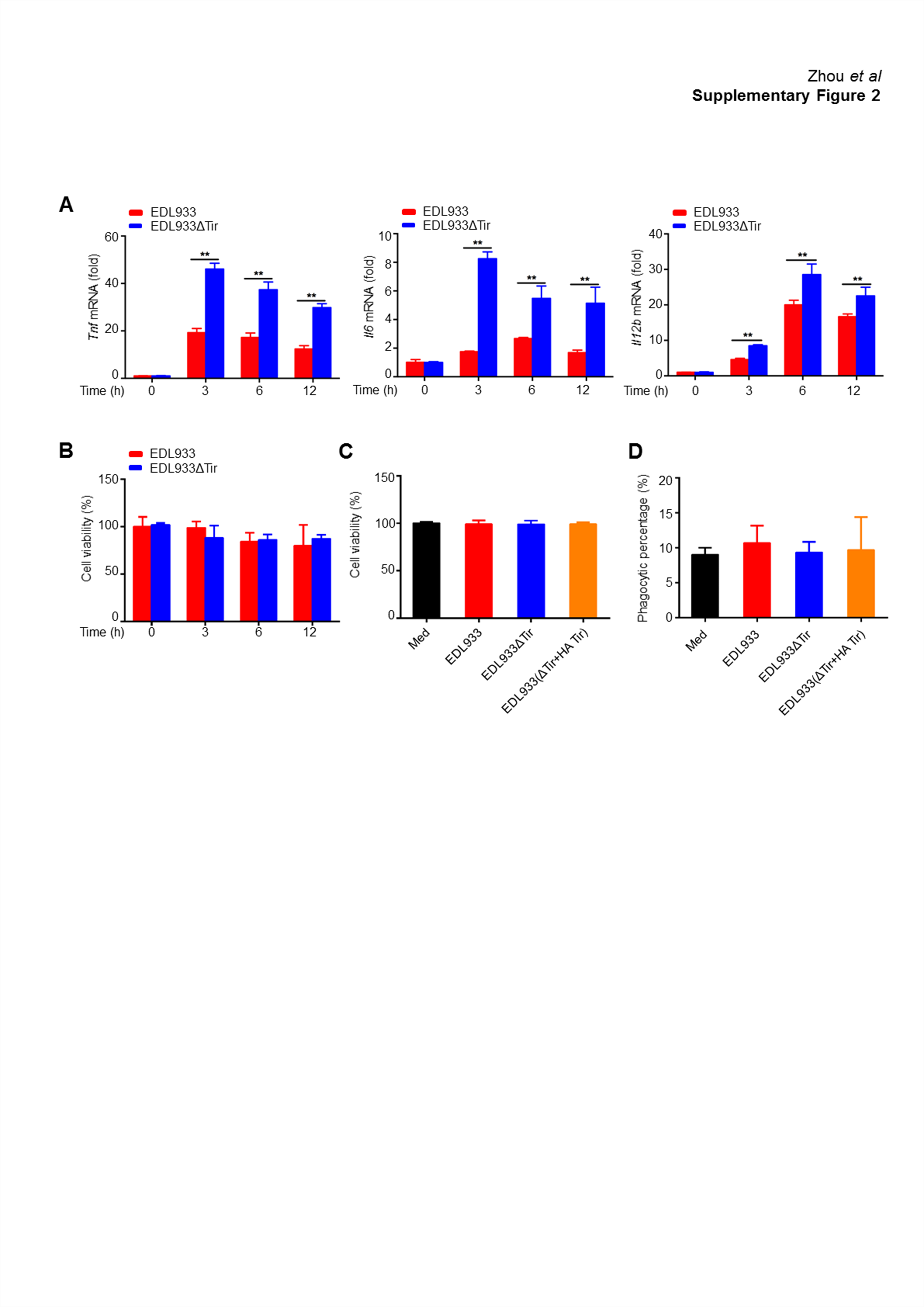


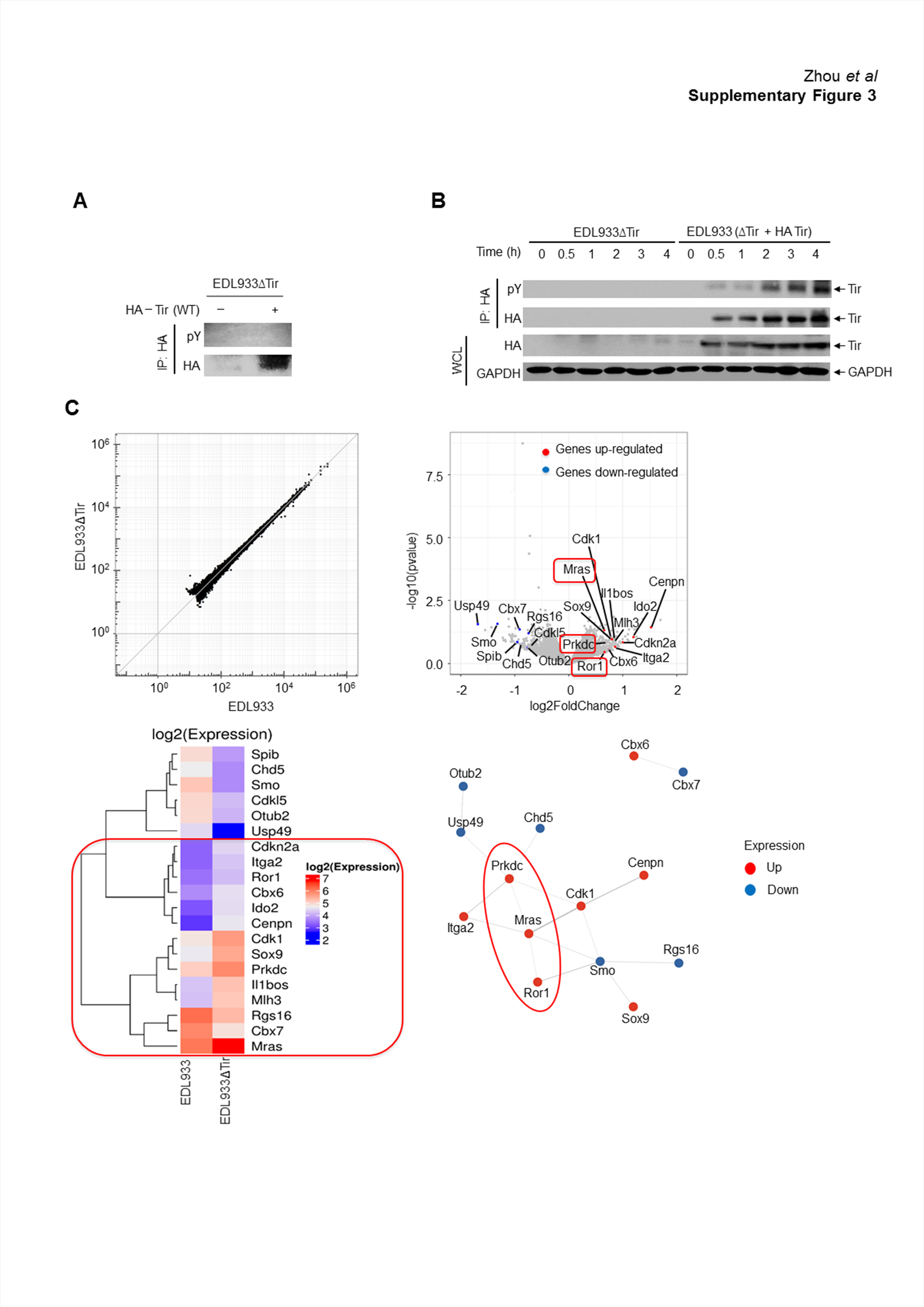


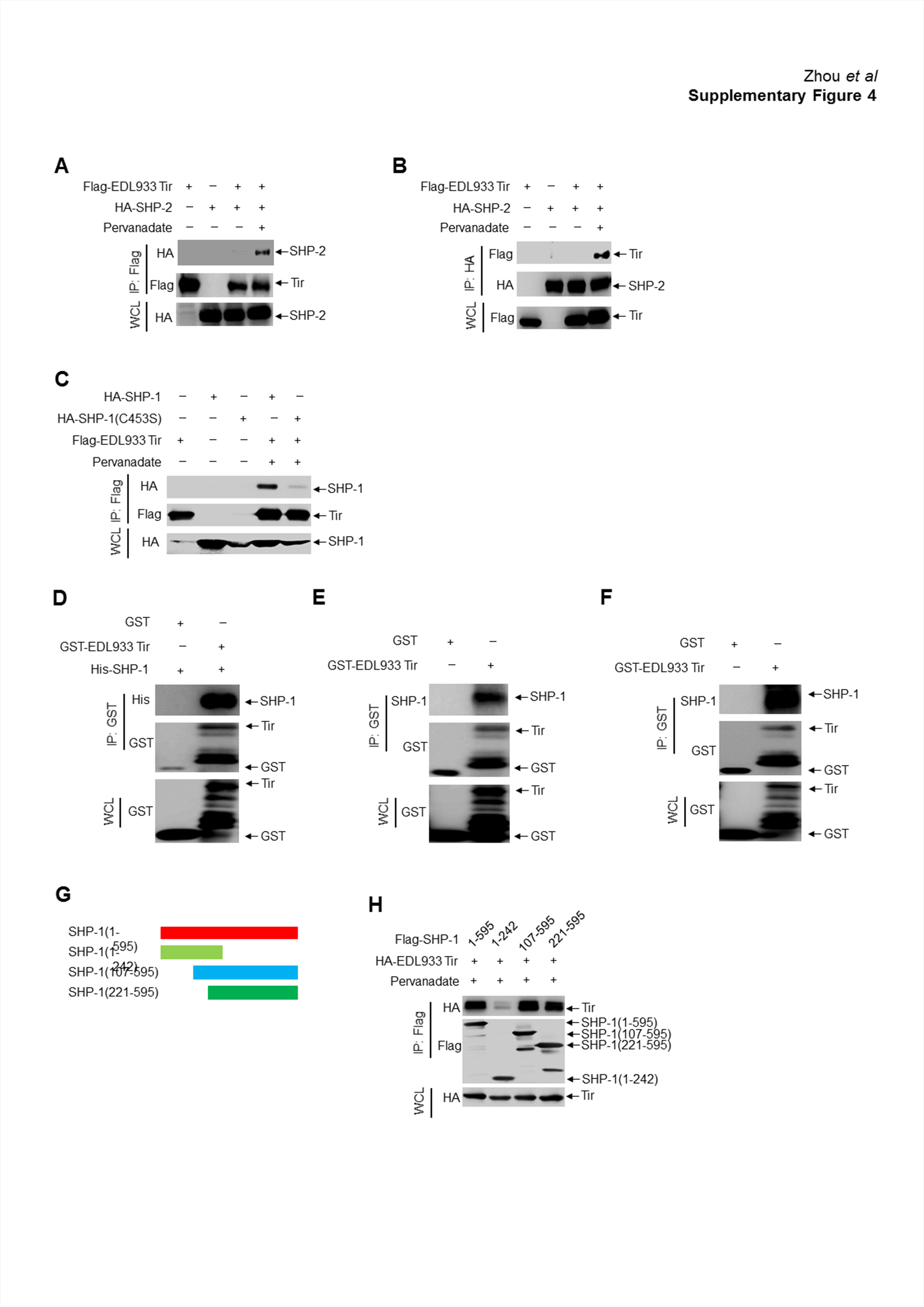


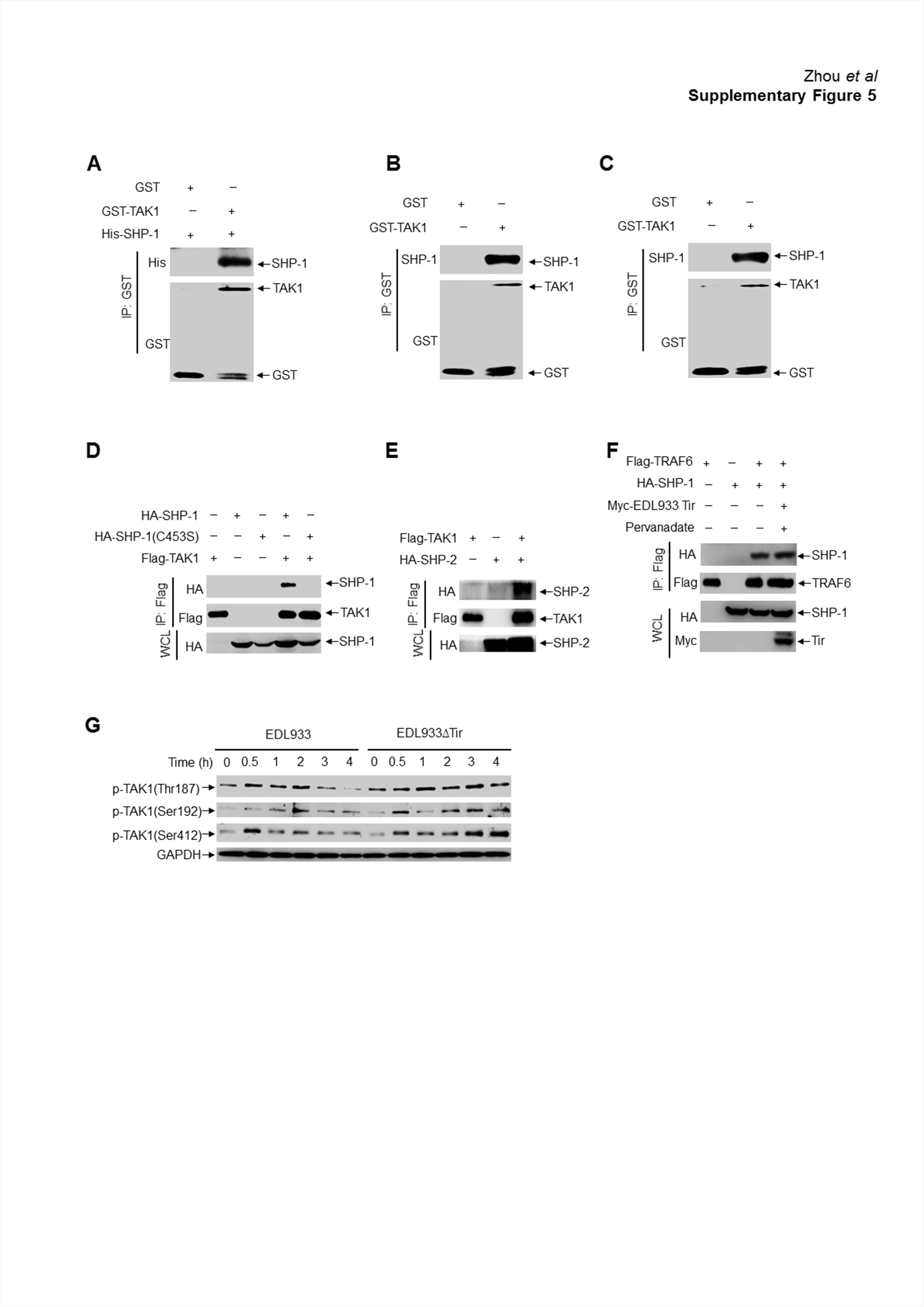


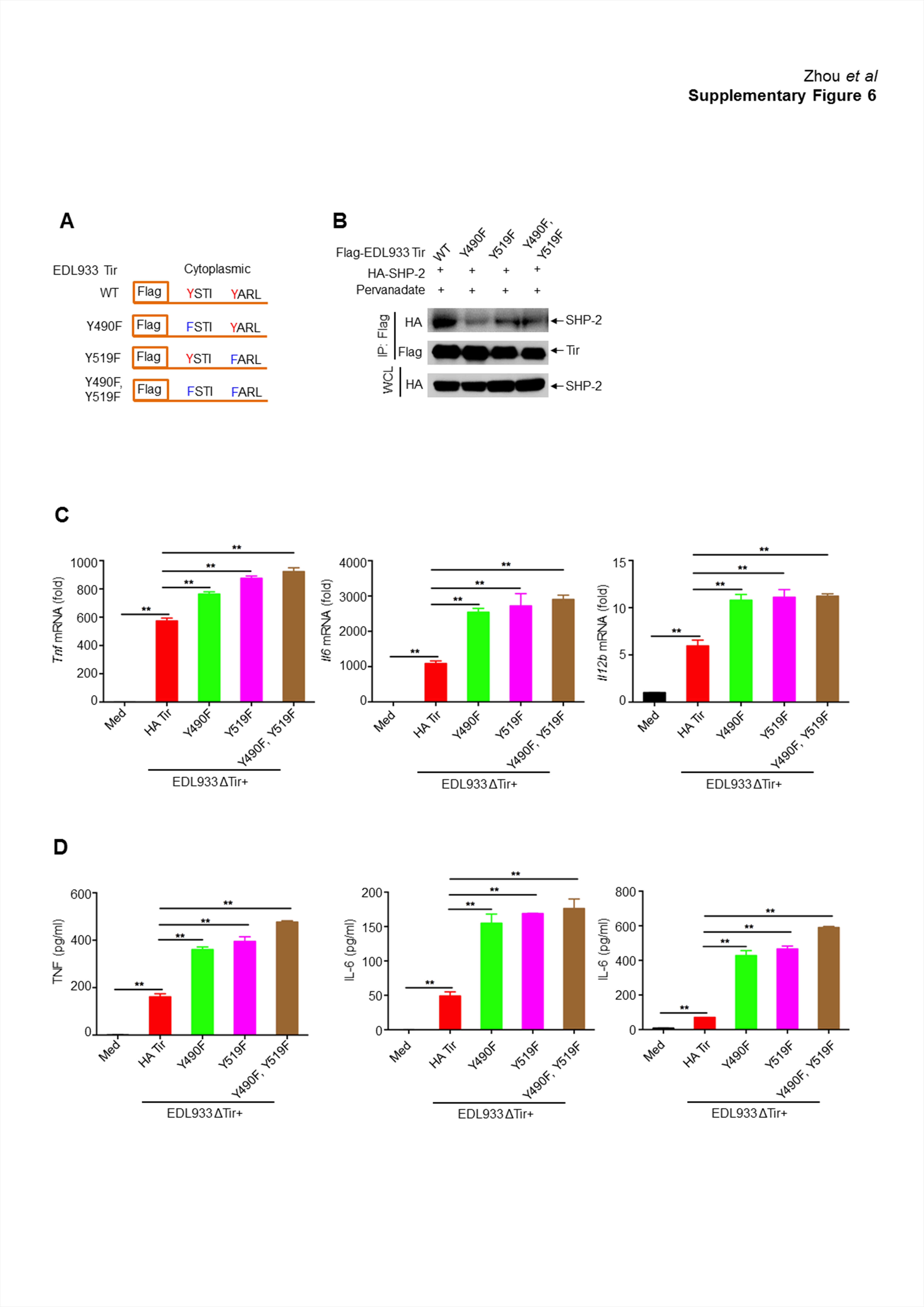


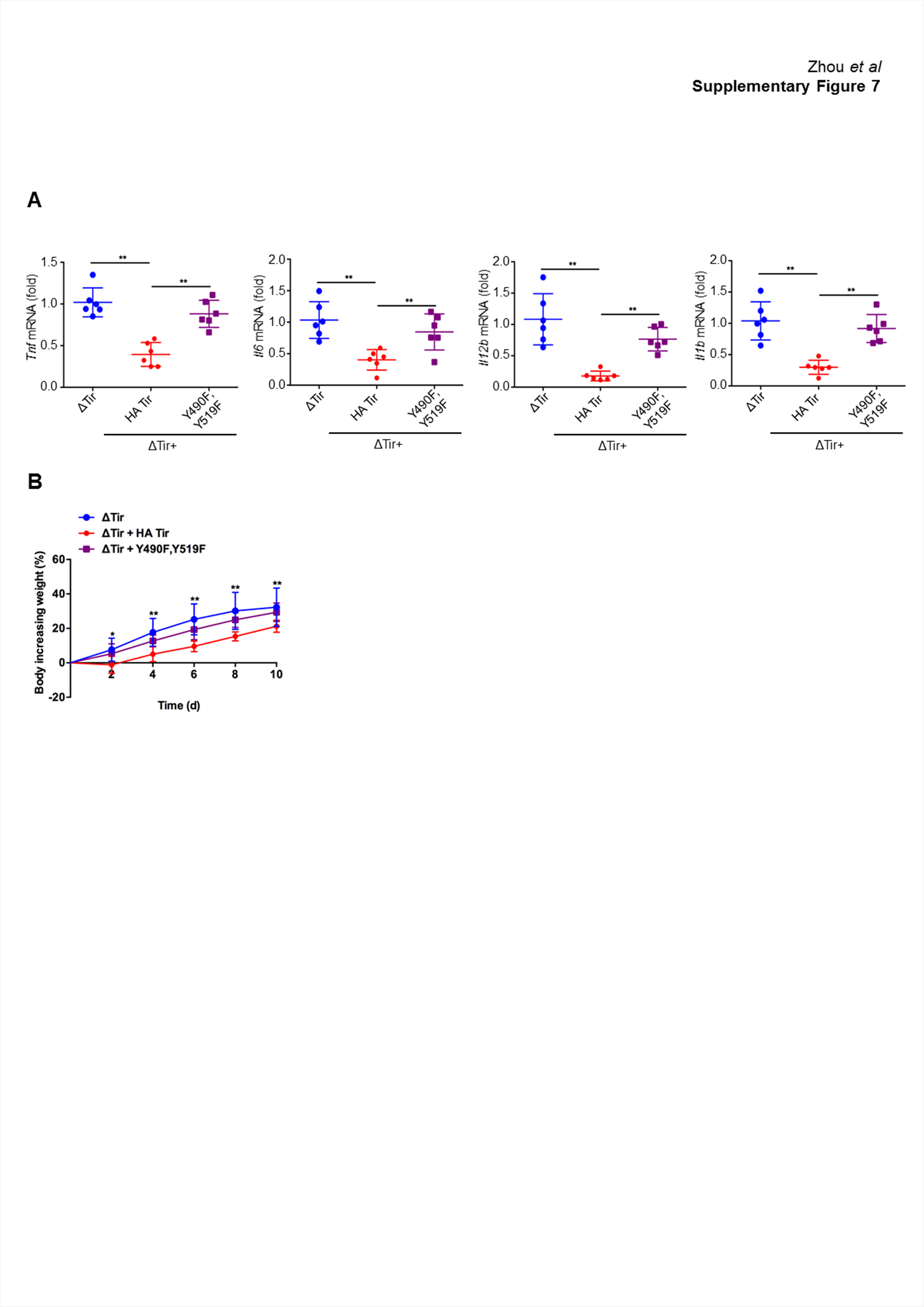


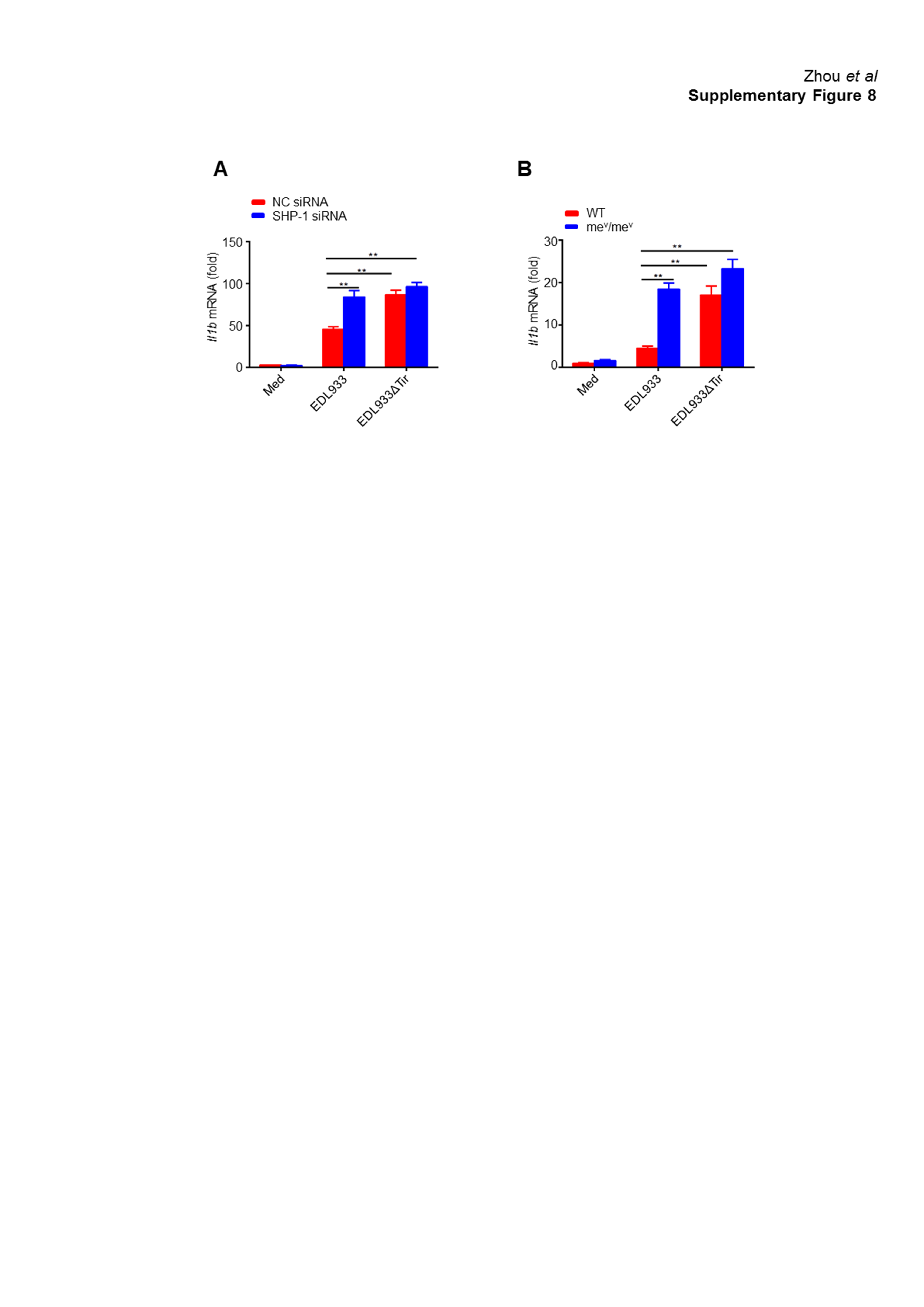


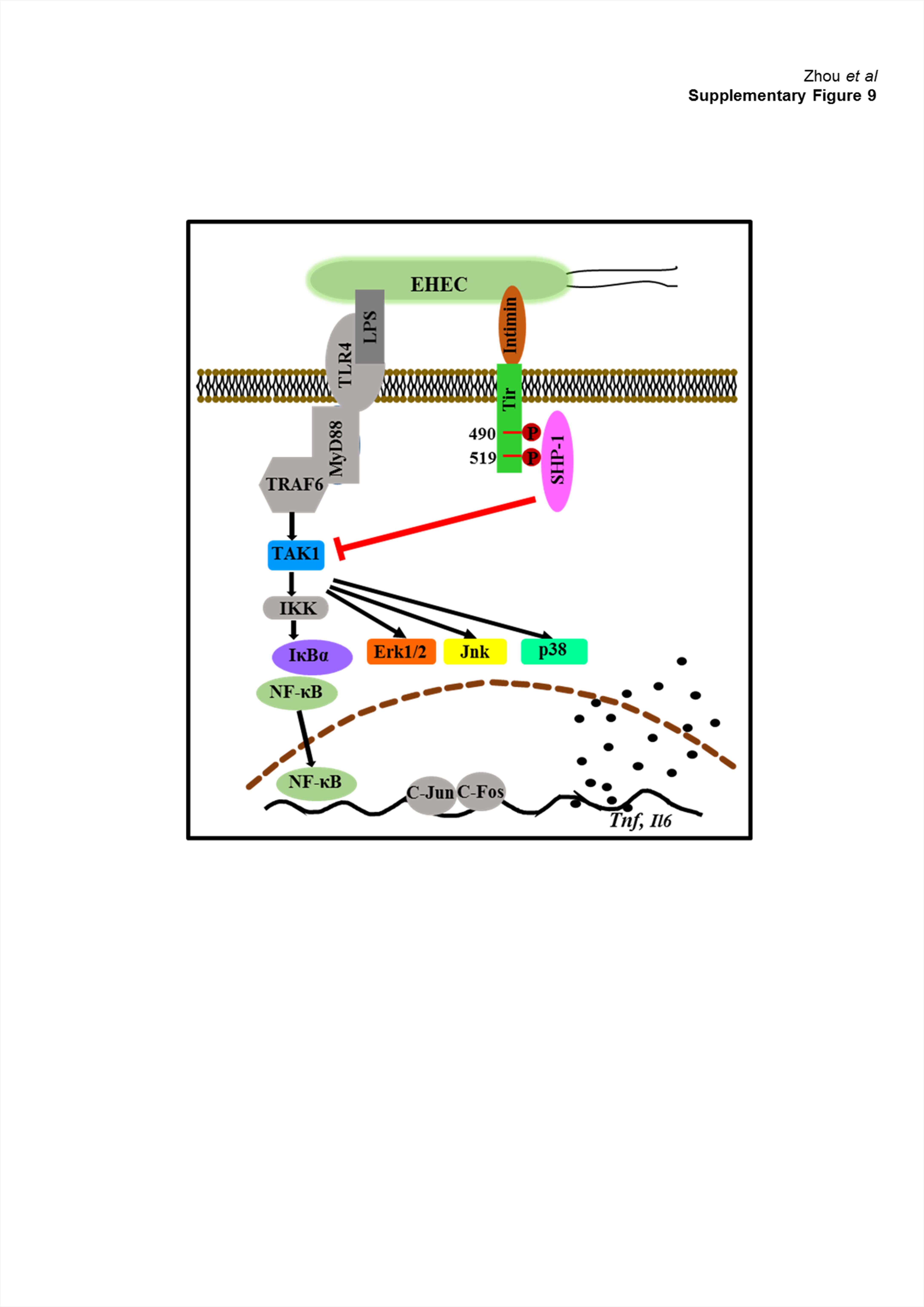

Supplement: Supplemental Material [file TEMI_A_1620589_SM7406.docx]
